# Whole-genome sequencing analysis of suicide deaths integrating brain-regulatory eQTLs data to identify risk loci and genes

**DOI:** 10.1038/s41380-023-02282-x

**Published:** 2023-10-04

**Authors:** Seonggyun Han, Emily DiBlasi, Eric T. Monson, Andrey Shabalin, Elliott Ferris, Danli Chen, Alison Fraser, Zhe Yu, Michael Staley, W. Brandon Callor, Erik D. Christensen, David K. Crockett, Qingqin S. Li, Virginia Willour, Amanda V. Bakian, Brooks Keeshin, Anna R. Docherty, Karen Eilbeck, Hilary Coon

**Affiliations:** 1 Department of Biomedical Informatics, University of Utah School of Medicine, Salt Lake City, UT USA; 2 Department of Psychiatry & Huntsman Mental Health Institute, University of Utah School of Medicine, Salt Lake City, UT USA; 3 Department of Neurobiology, University of Utah School of Medicine, Salt Lake City, UT USA; 4 Pedigree & Population Resource, Huntsman Cancer Institute, University of Utah, Salt Lake City, UT USA; 5 Office of the Medical Examiner, Utah Department of Health and Human Services, Salt Lake City, UT USA; 6 Clinical Analytics, Intermountain Health, Salt Lake City, UT USA; 7 Neuroscience Therapeutic Area, Janssen Research & Development, LLC, Titusville, NJ USA; 8 Department of Psychiatry, University of Iowa, Iowa City, IA USA; 9 Department of Pediatrics, University of Utah, Salt Lake City, UT USA

**Keywords:** Genetics, Neuroscience

## Abstract

Recent large-scale genome-wide association studies (GWAS) have started to identify potential genetic risk loci associated with risk of suicide; however, a large portion of suicide-associated genetic factors affecting gene expression remain elusive. Dysregulated gene expression, not assessed by GWAS, may play a significant role in increasing the risk of suicide death. We performed the first comprehensive genomic association analysis prioritizing brain expression quantitative trait loci (eQTLs) within regulatory regions in suicide deaths from the Utah Suicide Genetic Risk Study (USGRS). 440,324 brain-regulatory eQTLs were obtained by integrating brain eQTLs, histone modification ChIP-seq, ATAC-seq, DNase-seq, and Hi-C results from publicly available data. Subsequent genomic analyses were conducted in whole-genome sequencing (WGS) data from 986 suicide deaths of non-Finnish European (NFE) ancestry and 415 ancestrally matched controls. Additional independent USGRS suicide deaths with genotyping array data (*n* = 4657) and controls from the Genome Aggregation Database were explored for WGS result replication. One significant eQTL locus, rs926308 (*p* = 3.24e−06), was identified. The rs926308-*T* is associated with lower expression of *RFPL3S*, a gene important for neocortex development and implicated in arousal. Gene-based analyses performed using *Sherlock* Bayesian statistical integrative analysis also detected 20 genes with expression changes that may contribute to suicide risk. From analyzing publicly available transcriptomic data, ten of these genes have previous evidence of differential expression in suicide death or in psychiatric disorders that may be associated with suicide, including schizophrenia and autism (*ZNF501, ZNF502*, *CNN3*, *IGF1R*, *KLHL36*, *NBL1*, *PDCD6IP*, *SNX19*, *BCAP29*, and *ARSA*). Electronic health records (EHR) data was further merged to evaluate if there were clinically relevant subsets of suicide deaths associated with genetic variants. In summary, our study identified one risk locus and ten genes associated with suicide risk via gene expression, providing new insight into possible genetic and molecular mechanisms leading to suicide.

## Introduction

Suicide death is a major public health problem and leading cause of death [1]. Complex and heterogeneous risk factors for suicide death include environmental exposures, comorbid clinical conditions, and genetic variation [1–5]. Accumulated evidence suggests that genetic factors play a critical role in suicide risk, with heritability estimated to be 30–55% from twin and family studies [6, 7]. Thus, genetic investigations could advance our understanding of the biological basis of suicide risk, leading to development of more effective prevention strategies.

Well-powered large-scale genome-wide association studies (GWAS) have begun to identify genetic variants significantly associated with suicidal thoughts and behaviors including death [1, 8–10]. Additional independent GWAS studies have also identified several potential genetic susceptibility loci for suicidal behaviors in genes including *NCAN* [9] and *SOX5* [1] that are related to psychiatric conditions (e.g., schizophrenia and depression). Although GWAS have aided in identifying suicide-related genetic loci, how these identified loci contribute to suicide risk remains elusive [11].

Regulation of gene expression is critical for brain function [12, 13], with widespread dysregulated gene expression observed in psychiatric disorders associated with suicide [14–16]. For instance, a previous study reported that five key genes related to psychiatric diseases have decreased brain expression in individuals who died by suicide [17]. The vast majority of disease-associated genetic variants from human disease GWAS are located in non-coding regulatory regions, some of which may be associated with gene expression, which represent expression quantitative trait loci (eQTLs) [18]. That is, suicide-risk associated single nucleotide polymorphisms (SNPs) may play a significant role in risk of suicide by influencing gene expression in the brain as eQTLs, potentially leading to altered behavior or dysregulating other complex processes.

Integrative studies of GWAS and eQTLs have proven to be a powerful approach to identify novel genetic susceptibility loci with modest effects on various complex diseases [19–23]. The stringent significance thresholds required for GWAS to avoid detecting false positive genetic loci due to multiple testing limit genetic discovery to SNPs with small-to-moderate effects on complex diseases, potentially missing heritability [22]. Genomic association tests prioritizing eQTLs in regulatory regions can be useful in increasing analytic power and allowing discovery of actual mechanisms of risk through investigating only the subset of genome-wide SNPs that are associated with changes in gene expression [22]. The eQTL SNPs can play critical roles in complex trait phenotypes. Indeed, studies of psychiatric disorders integrating GWAS and eQTLs have successfully identified novel genetic loci that were not detected with GWAS alone (e.g., major depressive disorder (MDD) and schizophrenia) [20, 24–28]. Additionally, a recent study showed psychiatric disorders-related genetic variants are enriched at regulatory regions (e.g., histone modifications, DNA accessibility, and enhancer-promoter interaction regions affecting gene expression) [29] Although genomic studies integrating eQTLs in regulatory regions have been performed for several psychiatric disorders, to the best of our knowledge, this approach has not been taken for suicide death.

Here, we aim to identify novel regulatory suicide-associated genetic loci affecting gene expression by integrative analysis of multi-layer complimentary data, including genomic, transcriptomic, histone modification ChIP-seq, Hi-C, and clinical electronic health record (EHR) data. We initially obtained genome-wide brain-regulatory eQTLs from multiple available public resources. We then performed an association test of the eQTLs with suicide risk by analyzing genomic data generated from unrelated suicide deaths and ancestry-matched controls. In addition, we conducted a gene-based analysis to identify genes whose expression changes contribute to suicide risk [30]. Our study provides new insight into the genetic mechanisms of suicide.

## Materials and methods

**Fig. 1:**
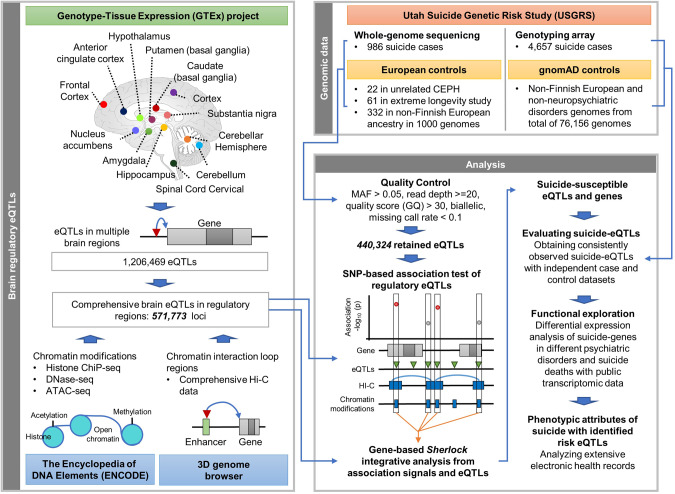
The study design for genomic analyses in comprehensive regulatory brain eQTLs. First, we identified 571,733 comprehensive brain eQTLs by integrating multi-layer data from different resources, as follows: (1) 1,206,469 eQTLs identified from 13 brain regions in Genotype-Tissue Expression (GTEx) project, (2) chromatin accessibility regions with histone modifications ChIP-seq, DNase-seq, and ATAC-seq in the Encyclopedia of DNA Elements (ENCODE) project, and (3) Hi-C data annotating enhancer from chromatin interaction loop regions in 3D genome browser. The whole-genome sequence (WGS) data from 986 suicide deaths of non-Finnish European (NFE) ancestry and 415 ancestrally matched controls was analyzed. Independent suicide deaths with genotyping array data (*n* = 4657) and controls from the Genome Aggregation Database were further investigated to replicate the results from WGS analysis. After quality control, a total of 440,324 eQTLs were retained to perform genetic association analysis at the individual-SNP level and at the gene level. For the gene-level analysis, integrative analyses were conducted using Sherlock. To explore evidence of dysregulated expression of identified genes in psychiatric disorders closely related with suicide risk, we explored two independent transcriptomic RNA-seq datasets measured in the disorders: (1) Yale-autism spectrum disorder (ASD) and UCLA-ASD studies in PsychENCODE for brain samples of ASD (*n* = 43) and ASD matched healthy control (*n* = 65) and BrainGVEX, CommonMind Consortium (CMC), and CMC-HBCC studies in PsychENCODE for brain samples of European ancestry individuals with bipolar disorder (BD, *n* = 145), schizophrenia (SCZ, *n* = 346), and healthy controls (*n* = 559), and (2) Korean mental health (KMH) genomics study for whole-blood samples of individuals with major depressive disorder (MDD, *n* = 39), suicide attempt (SA, *n* = 56), and healthy controls (*n* = 87). We further investigated transcriptomic datasets of suicide deaths: (1) GEO id: GSE66937 including 10 suicide deaths and 7 controls for each of three brain regions: amygdala, prefrontal cortex, thalamus, and 9 suicide deaths and 7 controls for hippocampus region and (2) GSE101521 including 21 suicide deaths and 29 controls. Finally, we merged electronic health record (EHR) data of ascertained samples for genomic analysis to identify specific co-occurring diagnostic phenotypes in suicide associated with identified risk loci.

An overview of the research design is illustrated in Fig. 1. The comprehensive brain eQTLs within regulatory regions (e.g., enhancer, promoter, and gene body) were obtained by systematically integrating multi-layer biological data including histone modification ChIP-seq data (e.g., H3K4me3), ATAC-seq, Hi-C data, and eQTL resources. WGS and clinical EHR data further were employed to identify suicide-associated SNPs acting as regulatory eQTLs and to evaluate their clinical attributions.

### Utah suicide death cohort ascertainment

The Utah Suicide Genetic Risk Study (USGRS) has a sample of >8000 DNAs from population-ascertained suicide deaths. Suicide deaths have been ascertained through a long-term collaboration with the centralized statewide Utah Office of the Medical Examiner (OME). DNA has been extracted from whole blood by using the state-of-the-art methods (https://ctsi.utah.edu/cores-and-services/ctrc/dna-extraction-facility). This study is approved by Institutional Review Boards from the University of Utah, Intermountain Health, and the Utah Department of Health and Human Services.

### Phenotypic electronic health records (EHR) data

Identifiers from suicide deaths were securely transferred from the OME directly to personnel at the Utah Population Database (UPDB, https://uofuhealth.utah.edu/huntsman/utah-population-database). The UPDB is a state-wide database that contains records on over 12 million individuals, including demographics, two decades of health records data, and deep genealogical data. After linking suicide deaths, identifiers were stripped before data were given to the research team to protect privacy and confidentiality. Linked diagnostic electronic health records were from statewide inpatient and ambulatory care encounters through Utah State Health Department records in addition to data from outpatient encounters from the largest two clinical data providers in the state (University of Utah Healthcare and Intermountain Health), representing ~85% of the state’s outpatient encounters. The inpatient and outpatient International Classification of Diseases (ICD-9; https://www.cdc.gov/nchs/icd/icd9.htm and ICD-10; https://www.cdc.gov/nchs/icd/icd10cm.htm) codes were curated within the UPDB to eliminate duplication. For efficient characterization of diagnoses, we collapsed the diagnostic data into interpretable categories using hierarchical classification derived through expert clinical adjudication (Drs. Keeshin, Docherty, and Monson). For this study, we included categories with prior evidence for association with suicide risk (alcohol related disorders, asthma, anxiety, neurodegenerative disorders, bipolar disorder, depression in a broad and narrow sense, all drug related disorders, specific opioid misuse, eating disorders, schizophrenia, pain, sleep disorders, and suicidal ideation).

### Whole-genome sequence data of suicide deaths and controls

WGS data was generated on 1053 Utah suicide deaths by using Illumina NGS technology with an average read depth of at least 20×. Alignment and variant calling and joint genotyping of suicide deaths and control WGS datasets was performed at the Utah Center for Genetic Discovery (UCGD) Core Facility, part of the Health Sciences Center Cores at University of Utah. The UCGD pipeline called variants using the Sentieon software package [31] which incorporates GATK best practices [32]. Sequence reads were aligned to GRCh38 (Genome Reference Consortium Human Build 38) using BWA-MEM (Burrows-Wheeler Aligner) [33]. The Haplotyper algorithm in Sentieon was used to produce genomic Variant Call Format (gVCF) files. Suicide death gVCF files were combined and jointly genotyped with 1241 control samples from three sources. 622 individuals were from the 1000 Genomes Project cohort (1000G) [34]. Five hundred and twelve individuals were from multigenerational Centre d’Etude du polymorphisme humain (CEPH) families [35]. Ninety-six individuals were from a study of longevity of healthy elderly individuals form Utah [36]. The final VCF file with suicide deaths and controls was recalibrated to limit false positive calls.

### Ancestry estimation and sample relatedness

We confined our analyses to unrelated suicide deaths and controls that had estimates of at least 90% non-Finnish European (NFE) ancestry. This threshold represents a conservative ancestry estimate as most USGRS samples are predominately European. We estimated the ancestry of the samples as a composition of five ethnicities (European, African, East Asian, Native American, South Asian) using the 1000 Genomes Project data (https://www.internationalgenome.org/data/) as a reference. We used a modified version of the pipeline presented by Giulio Genovese at https://github.com/freeseek/kgp2anc. First, our dataset was combined with the 1000G phase 3 dataset. SNPs were then pruned using the “--indep-pairphase” command in plink 1.9 [37]. PCA was run on the set of pruned SNPs with plink 2.0 [38]. Using the known estimated ancestry for AMR [34] and presumed ancestry for most other samples as the basis, we estimated the ancestry of every other sample as a combination of the 5 known ancestries using linear regression on the space of top 10 PCs with Mahalanobis distance defined by those top 10 PCs. Estimates of pairwise identity by descent (IBD) were calculated using Plink 1.9. Pairs of related individuals (third degree or closer) were identified with pi-hat values greater than 0.12. One member of each of the identified related pairs was randomly removed. After filtering our dataset included 986 suicide deaths and 415 control samples (1000G 332, longevity 61, CEPH 22).

### PsychArray genotyping data for confirmation analyses

Additional independent suicide deaths (*n* = 4657) were genotyped using the Illumina Infinium PsychArray platform (https://www.illumina.com/techniques/microarrays/array-data-analysis-experimentaldesign/genomestudio.htm), which assesses 593,260 single nucleotide polymorphisms (SNPs). Generation, processing, quality control and imputation of genotyping array data from suicide deaths in USGRS has been previously described [1, 5, 9]. We explored the imputed array data to confirm the results of our genomic analysis with WGS data using analysis methods described below.

### Brain eQTL data

Comprehensive brain eQTL data analyzed in this study were derived from the GTEx database (Supplementary Table S1). GTEx is a public resource for the study of gene expression and its regulation by analyzing WGS, whole-exome seq, and RNA-seq [39]. It provides a comprehensive eQTL resource observed from 54 healthy tissue sites from approximately 1000 individuals throughout the human body, including the brain. More detailed information of these data is described in the original study. We considered statistically significant eQTLs according to the criterion of adjusted *p*-value with false discovery rate (FDR) < 0.05 for each of 13 brain regions as described in Fig. 1.

### Annotation of regulatory regions

To obtain eQTLs in regulatory regions, we integrated 13 histone modification ChIP-seq (i.e., H2AFZ, H3F3A, H3K27ac, H3K27me3, H3K36me3, H3K4me1, H3K4me2, H3K4me3, H3K79me2, H3K9ac, H3K9me2, H3K9me3, and H4K20me1), ATAC-seq, and DNase-seq data processed by Encyclopedia of DNA Elements (ENCODE) project [40]. We first searched and downloaded experimental result data in a bed file format for narrow peaks observed from the histone modification data of the human brain described in the ENCODE project. These peaks include chromatin structure dynamic information that refers to regulatory regions. Furthermore, we combined high-throughput chromosome conformation capture (Hi-C) data that capture genome-wide chromatin interactions in cell nuclei to annotate enhancer regions that are regulatory regions distal from transcription start sites [41]. We obtained the comprehensive Hi-C data results of various cell types including the brain from the 3D genome browser. This browser collects independent studies on chromatin conformation (Hi-C) data [42]. Finally, we annotated robust regulatory regions by overlapping ENCODE histone modification peaks and enhancer regions. We included eQTLs within these annotated regulatory regions as an association test set in this study.

### Single genetic association test

Our primary analysis in this study was with WGS data. Although this includes a smaller number of samples compared to the genotyping array data, WGS data provides much higher resolution and covers nearly all possible eQTLs, such as those in regulatory regions, compared with array data. Unconditional generalized logistic regression model (GLM) was formulated to test for variant association with suicide death for each eQTL from WGS data, estimating *p*-values, odds ratio (ORs), and 95% confidence intervals (CIs) by using R. This association test was performed using an additive effect model, adjusting for sex and ancestry principal components (PCs) to account for possible residual effects of population stratification and genomic relatedness. We tested only eQTLs with biallelic genotypes and minor allelic frequency (MAF) > 0.05. We eliminated any eQTLs where genotypes were missing in >10% of individuals (missing call rate > 0.1 were excluded). Furthermore, for each association test, we retained genotypes only from individuals with average read depth *≥* 20 and genomic quality score (GQ) > 30.

After association tests for all eQTLs, we obtained significant index eQTLs with a statistical criterion (FDR < 0.1) after LD clumping that retained eQTLs with the lowest *p*-value in each linkage disequilibrium (LD; r2 *≥* 0.6) block. Next, to verify eQTLs associated with suicide death, we additionally explored genotyping array data from independent USGRS suicide deaths and an independent control sample from the Genome Aggregation Database (gnomAD; v3.1.2) [43]. GnomAD contains aggregated frequency data from various large-scale WGS reference studies including 76,156 whole genomes [43]. We assessed if (1) allele frequencies from the array data of suicide-eQTLs identified by WGS data were consistently different between suicide deaths and controls, (2) suicide-eQTLs which were found from both WGS and array data were also consistently replicated using gnomAD control frequency data. Since gnomAD provides only allele frequencies of the aggregated WGS data, individual genotypes and demographic information were not available from this source. The frequencies in gnomAD were calculated from individuals of non-Finnish European ancestry, selecting for those deemed as non-neuropsychiatric (NFE-NN) to avoid possible confounding originating from data from individuals of other ancestry and/or from individuals with neuropsychiatric conditions.

### Gene-based analysis using *Sherlock* integrative analysis

We performed genomic analyses to identify suicide-associated eQTLs in regulatory regions that potentially confer suicide risk by affecting gene expression of their gene targets. The *Sherlock* integrative framework explores potentially causal relationships between gene expression affected by eQTLs and disease. This strategy has previously identified novel gene associations with psychiatric disorders [15, 44]. The method integrates summary-based results of eQTLs and SNP association signals from genomic analyses through a Bayesian statistical framework. We utilized the *Sherlock* integrative analysis to further evaluate suicide risk-gene expression affected by eQTLs through integrating our genetic association and GTEx eQTLs results. For each gene, the *Sherlock* integrative analysis tool provides a score as LBF (logarithm of Bayes factor, which estimates the probability of a gene-suicide relationship) and *p*-value. A positive LBF indicates that a specific gene affected by eQTLs is likely associated with suicide risk, while a negative LBF suggests that the gene does not have an association. For each genomic analysis result from WGS and array data, we comprehensively identified genes associated with suicide based on the criteria of LBF > 0 and *p* < 5e−3. We then defined only HUGO protein coding genes where our results replicated across WGS and array data.

### Expression analysis of suicide susceptibility genes

The *Sherlock* integrative analysis method discovers trait-associated genes that have a predicted causality through the linkage between gene expression changes and suicide risk. Therefore, gene expression analysis of suicide deaths compared with control samples could theoretically allow us to verify the genes identified by this gene-based analysis.

There are RNA-seq datasets measured from different psychiatric disorders generated by two independent datasets: (1) PsychENCODE [45, 46] including brain samples of autistic individuals (autistic, *n* = 43) and non-autistic matched controls (*n* = 65) and of Caucasian individuals with bipolar disorder (BD, *n* = 145), schizophrenia (SCZ, *n* = 346), and BD-SCZ matched controls (*n* = 559), (2) Korean mental health (KMH) disorder genomics study [16] for whole-blood samples of individuals with major depressive disorder (MDD, n = 39) and suicide attempters (SA, *n* = 56), and healthy controls (*n* = 87) (Supplementary Table S2). PsychENCODE provides a public resource of transcriptomic data by aggregating RNA-seq generated from different projects. We analyzed the ASD and its matched control data generated from UCLA-autism spectrum disorder (ASD) and Yale-ASD projects, and BD, SCZ, and their matched control data generated from BrainGVEX, CMC, and CMC-HBCC projects. For PsychENCODE expression data, we downloaded and analyzed the normalized expression matrix file based on fragments per kilobase of exon per million mapped fragments (FPKM) values that are provided from the PsychENCODE database. For the second dataset (KMH), we obtained raw fastq files of all samples which were individually mapped to the human reference genome (GRCh38). Next, gene expression was estimated as TPM values by using RSEM (v.1.3.0) [47]. After that, we compared expression levels for each group with controls: ASD *vs*. control, BD *vs*. control, SCZ *vs*. control, MDD *vs*. control, and SA *vs*. control by using logistic regression with sex and age as covariates. Project study variables (e.g., BrainGVEX and CMC) were additionally considered as a covariate to avoid a potential bias from different studies. We defined statistical significance for differential expressed genes with FDR < 0.05.

In addition, we investigated transcriptomic expression datasets measured from brains of individuals that died from suicide generated by two independent cohorts (Supplementary Table S2): (1) transcriptomic array data measured from four different brain regions of suicide deaths and decreased controls; 10 suicide deaths and 7 controls for each of amygdala, prefrontal cortex, and thalamus regions, and 9 suicide deaths and 7 controls for hippocampus region (GEO id: GSE66937) and (2) RNA-seq data of suicide deaths (*n* = 21) and controls (*n* = 29) (GEO id: GSE101521 [14]). For the array data, we downloaded and analyzed normalized expressions. For the RNA-seq data, data were processed with the GRCh38 human reference genome using the same methods as with the KMH dataset, described above. Due to the relatively small sample size, we considered significant differentially expressed genes to be those with *p*-value < 0.05 as determined empirically through 1000 repeated randomizations of the data.

### Investigation of demographic and phenotypic characteristics of suicide death samples with suicide-risk genetic variants

To further evaluate if there were clinically relevant characteristics in suicide deaths associated with identified genetic variants, such as a specific suicide subtype, we explored the International Classification of Diseases (ICD) diagnostic codes (ICD-9/ICD-10) in EHR data of our analyzed individuals who died from suicide. Details of cohorts that have EHR data are presented in Table 1. We characterized psychiatric phenotypes by aggregating ICD codes in EHR data as previously described [9] for relevant exposures and psychiatric diagnoses. We compared demographic and diagnostic information between suicide deaths with and without any of the genetic findings identified from the previous analyses.

**Table 1:** Demographic and clinical information for Utah suicide deaths analyzed in this study.

Affection status	Suicide deaths	
	WGS data	Array data
*N*	986	4657
*N* of electrical medical records	936	4001
Demographic information		
Female, *n* (%)	274 (28.1)	1,015 (21.8)
Age, mean (SD)	32.1 (13.4)	41.5 (17.5)
Clinical characteristics, *n* (%)		
Alcohol related disorders	269 (28.7)	1002 (25)
Asthma	154 (16.4)	497 (12.4)
Anxiety	493 (52.6)	1618 (40.3)
Bipolar disorder	396 (42.3)	567 (14.1)
Broad depression	593 (63.3)	2047 (51.0)
Dementia (Neurodegenerative)	128 (13.6)	493 (12.5)
All drug related disorders	369 (39.4)	1141 (28.4)
Eating related disorders	21 (2.2)	47 (1.1)
Major depressive disorder	366 (39.1)	1216 (30.3)
Obesity	192 (20.5)	865 (21.5)
Opioid misuse	171 (18.2)	518 (12.9)
Pain	651 (69.5)	2802 (69.9)
Schizophrenia	59 (6.3)	116 (2.8)
Sleep related disorders	276 (29.4)	1132 (28.2)
Suicidal Ideation	279 (29.8)	754 (18.8)

Demographic and clinical characteristics are described separately for suicide deaths with whole-genome sequencing (WGS) data and those with array data. Prevalence of clinical diagnoses from linked diagnostic electronic health records data is given for categories with known associations with suicide risk.

### Sex differences

Since gene expression differences in brain in psychiatric phenotypes and suicide deaths have been characterized by substantial sex differences [48], we performed a secondary expression analysis stratified by sex in two psychiatric disorder datasets and two SD datasets to identify additional differentially expressed genes in females vs. males, specifically. We defined male-specific genes as those with FDR < 0.05 in males but > 0.05 in females, and female-specific genes as those with FDR < 0.05 in females but > 0.05 in males.

## Results

A detailed summary of the suicide death cohorts analyzed in this study is provided in Table 1. The controls with jointly called WGS (*N* = 415) were unrelated adults of European ancestry, and were 51.1% female. Controls were ascertained for absence of major psychiatric disease.

### Individual eQTL association analysis

As shown at the Fig. 1 and supplementary Table S1, we identified a total of 1,206,469 eQTLs significantly associated with 17,976 target gene expression levels (FDR < 0.05) in multiple brain regions from the GTEx resource. Among them, 717,852 eQTLs were located in comprehensive chromatin accessibility (e.g., open) regions from different histone modification ChIP-seq, DNase-seq, and ATAC-seq, or were eQTLs that fell within chromatin interaction regions from Hi-C datasets. We then identified 571,773 eQTLs that overlapped between ENCODE and Hi-C data as potentials for our genomic association analysis test set. Among these regulatory brain eQTLs, 440,324 eQTLs passed the quality standards based on MAF, read depth, GQ, and call rate (See the Materials and Methods) with WGS data, resulting in our final eQTL association test set. Information on the proportions of eQTLs across all brain regions is presented in Supplementary Table S3.

**Fig. 2:**
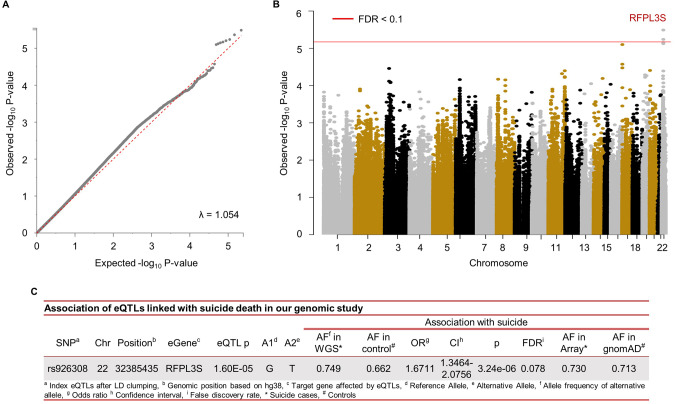
Individual-SNP-based associations with suicide in a genome-wide WGS eQTL analysis. **A** the quantile-quantile plot with *p*-values of our genomic analysis. *Y*-axis refers observed -log_10_ of *p*-values and *X*-axis indicates expected −log_10_ of *p*-values under H0. **B** Manhattan plot describing SNPs associated with suicide in our analysis. *Y*-axis and *X*-axis reflect observed −log10 of *p*-values and chromosomes, respectively. Red line indicates FDR corrected statistical significance (FDR < 0.1). **C** One index eQTL was detected with our significant criteria of FDR < 0.1.

After LD clumping, there were 46,075 index eQTLs that had the strongest associations with suicide for each LD block. Four eQTLs met our criteria for statistically significant associations with suicide death based on multiple testing correction (FDR < 0.1) from WGS genomic analysis. Further analyses combining genotyping array data from independent USGRS suicide death samples and gnomAD controls were performed to control potential bias from differences in molecular platforms. These analyses eliminated three eQTLs, with one remaining significant eQTL (Fig. 2; rs926308, chr22: 32385435, *p* = 3.24e−06): the three eQTLs were removed in Fig. 2. The QQ (quantile-quantile) plot and Manhattan plot from the WGS eQTL association test is depicted in Fig. 2, showing a genomic inflation factor (λ) of 1.054.

**Fig. 3:**
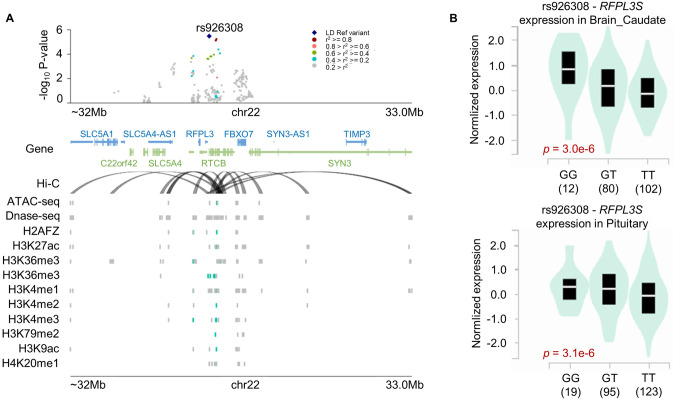
Results of rs926308 (chr22:32385435) identified by our SNP-level association study. **A** A regional plot for rs926308 on chromosome 22. The Hi-C line refers to chromatin interaction loops. Other lines indicate chromatin accessibility regions observed from histone modifications ChIP-seq, DNase-seq, and ATAC-seq data. Cyan colors are ChIP-seq peak regions overlapped in SNPs within the LD block of rs926308, while gray areas do not. **B** A violin plot of RFPL3S gene expression according to the genotypes of rs926308 generated from the Genotype-Tissue Expression (GTEx) project.

Variant rs926308 lies within an LD block with FDR = 0.078 (*p* = 3.24e−06) with an odds ratio (OR) per alternative allele *A* = 1.67, 95% CI: 1.35–2.08 (Fig. 2C). A regional plot of this SNP is depicted in Fig. 3A. GTEx data (Fig. 3B) shows that the risk allele rs926308-*T* decreases *RFPL3S* expression levels in pituitary (*p* = 3.1e−6) and caudate (*p* = 3.0e−6).

### Genes leading to suicide risk through *Sherlock* analysis integrating eQTLs and WGS results

Our study was performed under the assumption that gene expression changes could confer suicide risk, and since one gene expression perturbation could be associated with multiple modest effect eQTLs, gene-based analysis collecting multiple genetic variants could uncover further novel genes that have a putative role in suicide risk. To infer genes whose expression may contribute to suicide risk, we utilized *Sherlock* integrative analysis to systematically integrate summary-based results of SNP associations from our genomic analyses and eQTLs in multiple brain regions from GTEx. We considered genes to be potentially significant for suicide risk when the genes were replicated in *Sherlock* analyses with results from WGS analysis and genotyping array analysis. Using this approach, we identified a total of 20 genes that consistently resulted from both analyses (Supplementary Table S4). That is, for each gene, at least one eQTL is associated not only with altered gene expression but also with suicide risk simultaneously, suggesting that the eQTLs could contribute to suicide risk by affecting their target gene expression (Supplementary Table S4).

### Differential expression analysis

The 20 genes that we found from integration of association results and eQTL results were identified under the inference that dysregulation of their expression could potentially have a role in suicide risk. We investigated the expression of the 20 genes to find additional lines of evidence that expression changes could be related to conditions associated with suicide risk. There are publicly available RNA-seq datasets measured from different psychiatric disorders (e.g., ASD, BD, SCZ, MDD, and SA). Therefore, we assessed differentially expressed genes by analyzing those datasets to find evidence of potential roles of the 20 genes, since psychiatric disorders could be potentially associated with suicide risk. We compared gene expression between psychiatric disorders and healthy controls generated from two independent studies and between suicide deaths and controls from two additional independent studies (See the Materials and Methods section).

**Fig. 4:**
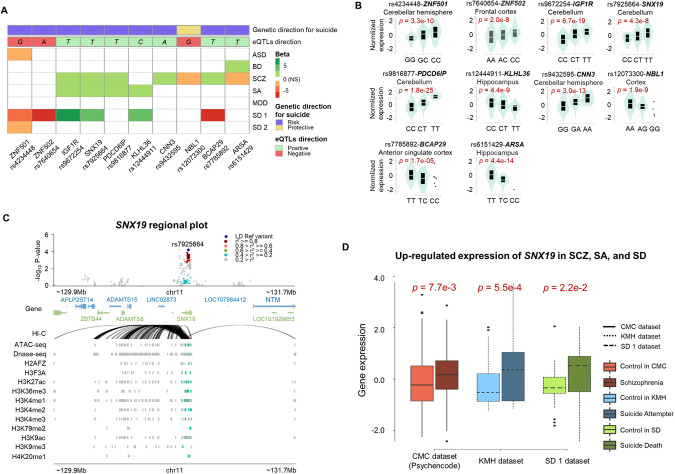
Significantly differentially expressed genes among the suicide-associated genes observed from *Sherlock* integrative analysis. **A** A heatmap of differential expression in each psychiatric disorders compared to the controls. Red and green shading indicates downregulated and upregulated expression, respectively, in the given psychiatric disorders and suicide deaths. eQTL annotation above the heatmap represents the direction of each eQTL, with red and green reflecting negative and positive beta values between gene expression, respectively, and noting that the allele for the eQTL is indicated in the box. The genetic direction for suicide annotation represents the topmost line of the figure, with purple and yellow referring to the given eQTLs direction as a risk or protective allele, respectively. For example, for *CNN3* gene, eQTLs direction of *A* of rs9432595 (showed at **B**) is positive that expression tends to increase in allele *A* of the SNP, and this allele *A* was observed to be risk allele from our suicide WGS genomic analysis. **B** Violin plots of expression of the ten identified genes according to eQTLs generated from Genotype-Tissue Expression (GTEx) project. **C** A regional plot for *SNX19* on chromosome 11. The Hi-C plot demonstrates chromatin interaction loops. Other lines indicate chromatin accessibility regions observed from histone modifications ChIP-seq, DNase-seq, and ATAC-seq data. Cyan colors are ChIP-seq peak regions overlapped in SNPs within the LD block of rs7925664, while gray areas do not. **D** Boxplot demonstrating that *SNX19* was significantly upregulated in individuals with schizophrenia, suicide attempters, and suicide deaths from three independent RNA-seq datasets: (1) CommonMind Consortium (CMC) in PsychENCODE, (2) Korean mental health (KMH), and (3) suicide death dataset 1 (GEO id: GSE66937). *X*-axis and *Y*-axis refer data projects and normalized gene expression, respectively. ASD autism spectrum disorder, BD bipolar disorder, SCZ schizophrenia, SA suicide attempter, MDD major depressive disorder, SD1: Suicide death 1 (GEO id: GSE66937), and SD2: Suicide death 2 (GEO: GSE101521).

We identified nine genes that have significantly different gene expression in at least one of five different psychiatric disorders compared to the control groups (Fig. 4 and Supplementary Table S5); one additional gene, *ZNF501*, was suggestive (FDR = 0.058). For example, expression of *ZNF501* was observed to be decreased in ASD samples compared to controls (*p* = 7.64e−03). Expressions of *BCAP29* (2.34e−04), *CNN3* (1.59e−06), *IGF1R* (1.81e−07), *PDCD6IP* (5.5e−10), *SNX19* (1.65e−04) were increased in SCZ, while *NBL1* (2.46e−12) expression was decreased in SCZ. Furthermore, expressions of *IGF1R* (1.59e−04), *KLHL36* (2.91e−03), *PDCD6IP* (5.43e−03), and *SNX19* (1.48e−03) were all observed to be increased in SA. However, *ARSA* gene expression was observed to be increased in BD (3.62e−04), but was decreased in SCZ (4.50e−03). We further identified that *ZNF501*, *ZNF502*, *IGF1R*, *SNX19*, *KLHL36*, and *BCAP29* were differentially expressed in suicide death (SD) (Supplementary Table S6 and Fig. 4A). *ZNF502* was unique to SD, but the other five genes overlapped with the results from other psychiatric disorders above (Fig. 4A).

Therefore, we found additional evidence that expression changes in ten genes by eQTLs may have a role in molecular mechanisms that are underlying suicide risk potentially shared with risk of psychiatric disorders. Detailed results of the ten identified genes are provided in Supplementary Tables S5 and S6.

Of note, for each of seven genes (i.e., *ZNF501*, *ZNF502*, *CNN3*, *IGF1R*, *PDCD6IP*, *SNX19*, and *KLHL36*), expression regulation was shown to have a consistent direction in different layers of data from different resources. For instance, *SNX19* gene expression was observed to be increased in SCZ from PsychENCODE, SA from KMH, and SD from suicide death array dataset (Fig. 4A, D). In addition, the expression of *SNX19* tends to increase with allele *T* of rs7925664 (Fig. 4B), and our genomic analysis revealed that frequency of the rs7925664-*T* was observed to be significantly increased in suicide (Fig. 4C): OR = 1.54 [CI: 1.25–1.91] from WGS data and OR = 1.31 [1.13–1.52] from array data. That is, rs7925664 may act as a risk variant for suicide by increasing *SNX19* expression, with a similar pattern being observed in SCZ, SA, and SD.

### Sex differences

We identified three male-specific genes (*ARSA*, *IGF1R*, and *SNX19*) and two female-specific genes (*LEMD2* and *PCP4*) in psychiatric disorders (Supplementary Table S7). However, none of these genes were significant in SD datasets.

### Phenotypic attributes of suicide deaths with genetic variants

When comparing individuals who died from suicide with and without significant variation, as identified above, there was no significant difference in sex distribution or suicide death age. In addition, no different demographic or diagnostic variables achieved significance according to our criteria (FDR < 0.05) (Supplementary Tables S8 and S9). However, of note, we observed one suggestive association of lower prevalence of BP between suicide deaths with and without rs12444911 in both of WGS (*p* = 0.01) and array (*p* = 0.02) cohorts. Approximately 38.6% of individuals who died from suicide who had the alternative allele *T* had a BD diagnoses, while 46.1% of those with the reference allele had a BD diagnosis in the WGS cohort; 12.8% and 15.1% of deaths had a BD diagnosis with and without the allele *T* in the array cohort. That is, lower prevalence of BD was observed in suicide deaths with the allele *T* than those without the allele. Finally, neither age nor sex significantly impacted the relationships between genetic variants and EHR diagnosis.

Furthermore, we evaluated whether there are sex-specific associations of ICD diagnoses in suicide deaths with the sex-specific five identified gene eQTLs, as done with the ICD analysis presented above, but did not find sex-specific ICD diagnoses associated with the eQTLs (data not shown).

## Discussion

In this study, we analyzed USGRS WGS and genotyping array data in combination with rich regulatory data resources to identify new genetic loci and genes involved in risk of suicide. We prioritized brain-regulatory eQTLs with potential causal effects on the modification of brain functional gene pathways. Since suicide and associated psychiatric disorders are genetically complex, our strategy to discover eQTL SNPs with potentially interpretable effects on gene function is essential to understanding additional genetic factors contributing to suicide risk, which could be missed by GWAS, as has been identified in other integrative studies [22]. Results revealed one novel genetic locus impacting Ret Finger Protein-Like 3S (*RFPL3S*) gene expression. This result was also replicated through rigorous investigation within two independent suicide cohorts and two independent control resources. Additionally, we systemically integrated our genetic association results and brain eQTLs using a *Sherlock* integrative analysis, identifying 20 genes where expression changes may contribute to suicide risk. Further comparative transcriptomic analysis showed that ten of these 20 genes may also be dysregulated in other psychiatric disorders, with six being specifically identified within SD, providing potential pathways of gene expression perturbations in suicide risk.

The identified risk genetic variant, rs926308, is a *RFPL3S* eQTL where the alternative allele decreases *RFPL3S* gene expression (Fig. 3B) in a brain-specific manner (See Fig. S1). Since the rs926308 SNP is closer to other genes, such as *RTCB*, we investigated the GTEx dataset to see if this SNP is also associated with other gene expressions. We found significant associations with expressions of *SLC5A4* and *RTCB* in testis and colon, respectively, but no associations with any genes expressed in brain tissues (Fig. S2). *RFPL3S* is one of the family of Ret finger protein-like proteins that are critical in primate neocortical development [49, 50]. Moreover, *RFPL3S* is significantly associated with arousal [49], a domain that is part of the National Institute of Mental Health Research Domain Criteria (RDoC) framework [51, 52]. This domain encompasses broad aspects of arousal related to stress response and anxiety [53], as well as sleep-wake changes [54]; abnormalities in these areas are plausible contributors to suicide risk. Our results warrant further study of *RFPL3S*, including downstream effects and developmental consequences of altered *RFPL3S* expression, and exploration of regulatory changes in other genes related arousal. Finally, this result should be interpreted with some caution as the rs926308 allele frequency (AF) was noted to differ between our two control datasets: jointly called 415 general population controls (AF = 0.67) and gnomAD (0.712) controls. This difference may result from presence of subclinical psychiatric conditions or residual population structures. However, despite this observation, both WGS and array suicide showed consistent increased AF compared to these two control sets.

Our *Sherlock* integrative analysis found 20 genes where expression changes may contribute to suicide risk, with nine of these genes having evidence of differential expression in psychiatric conditions, including schizophrenia (SCZ), autism spectrum disorder (ASD), depression, stress response, and neurodegeneration. Specifically, findings implicated variants associated with overexpression of SCZ-associated genes: *SNX19* [55–58], *CNN3* [59, 60], *BCAP29* (opposite direction of prior findings [61]), and *IGF1R* (opposite direction of prior findings [62]). *ZNF501* was found to have diminished expression with the associated variant in this study, consistent with prior ASD [63] and depression studies [64]. *KLHL36* has been previously associated with stress response by gene-based analysis from a GWAS of psychological resilience self-assessed by questionnaire [65, 66]. *PDCD6IP* was previously observed to be upregulated in MPTP (1-methyl-4-phenyl-1,2,3,6-tetrahydropyridine) induced neurodegeneration in mouse models [67]. Perturbation of the regulation of any one of these genes could contribute to the disruption of key emotional regulation and expression, perceptual, or developmental processes that could lead to greater risk of suicidal behavior. However, further research will be required to identify specific outcomes and downstream effects from such altered expression in the developing brain and to identify how such changes might alter suicide risk.

Our expression analysis results implicating different psychiatric conditions may provide an insight into putative roles of identified susceptibility genes in suicide risk that is shared with these other disorders. However, since co-occurring psychopathology does not fully account for suicide risk, the other 11 genes that did not have differential expression in any psychiatric disorders can still be considered as candidates specifically for suicide risk. For example, *ZNF502* was observed to be significantly differentially expressed in SD data. Also, while *SLC18A2* does not have differential expression in other psychiatric disorders, it is one of several serotonergic genes that facilitates the transport of vesicles containing serotonin to the presynaptic neuron, a process that has been associated with suicidal behavior [68–70]. Also, *LEMD2* is associated with cognition through mediating neuronal activity [71]. That is, these genes may be associated with suicide risk via different mechanisms independent from the psychiatric conditions we investigated. Although these genes were not detected in the available SD gene expression datasets, expression analysis with larger SD sample sizes will be required to identify further evidence. In summary, our *Sherlock* integrative analysis and expression analysis provides evidence of putative susceptibility genes modulated by eQTLs and their pivotal role via dysregulated expression in suicide risk.

Analyses using *Sherlock* provide a powerful approach to explain the relationship between gene expression affected by eQTLs and a disease of interest by integrating results of eQTLs and genomic association analysis. This approach assumes co-occurrence between effects of eQTLs on gene expression and evidence of association of the eQTLs with a disease [30]. For example, to establish the fact that the expression of a gene indeed confers disease risk, all eQTLs targeting expression of the gene must also be associated with disease risk since the eQTLs can change the gene expression. If one of the eQTLs is not significantly associated with disease risk, this will negatively affect the score of a *Sherlock* integrative analysis, reflecting the result that gene expression controlled by that eQTL does not affect disease risk. Based on this assumption, the gene-based *Sherlock* integrative analysis calculates the score indicating a probability of relationships between gene expression affected by eQTLs and disease risk. This analysis allows identification of suicide-risk eQTL SNPs aggregated at the gene level although the individual eQTL SNPs may have not achieved genome-wide significance in the single SNP-level association test.

Our genomic analysis with the Utah suicide cohorts has several strengths. First, our study explored genomic data generated from confirmed suicide deaths, rather than from individuals with non-lethal suicide-related behaviors which pose measurement challenges and may reflect relatively distinct and more heterogeneous phenotypes. The unambiguous and more severe outcome of suicide death may increase statistical power to detect genomic associations [9]. In addition, previous studies of suicide risk often focus on suicidal behaviors among individuals with specific psychiatric diagnoses. While this design may reduce heterogeneity, generalizability is impacted. Our study of population-ascertained suicide deaths allows for the potential of more generalizable results.

Second, we analyzed two independent suicide death cohorts (WGS and genotyping array) that have different clinical characteristics resulting from selection criteria [72]. The WGS cohort was prioritized for suicide deaths with significant extended familial risk, a factor associated with significantly younger age at death, a higher percentage of female suicide deaths, and more co-occurring clinical diagnoses [72]. As shown at the Table 1, although the most prevalent co-occurring clinical phenotype of the suicide deaths in both cohorts was pain, a larger portion of the WGS cohort had bipolar disorder (42.3% vs. 14.1% in the array cohort) and suicidal ideation (29.8% *vs*. 18.8% in the array cohort). Our design included the confirmation of allele frequencies across these cohorts, requiring robust consistency in the face of known differences in phenotypic and genetic risk loading. Of note, the increased WGS extended familial risk has been shown to be associated with increased genetic liability [72]. Thus, the focus on WGS analysis in our study may have advantages for the investigation of the selected eQTL variants.

Third, our study had access to WGS data, with confirmation of allelic differences observed in genome-wide array data. Although the array cohort included more suicide deaths than the WGS cohort, WGS data covered the entire genome for genetic variations. In addition, we prioritized brain eQTLs in regulatory regions unlikely to be covered in array data. Indeed, previous studies have reported that the WGS genotype calling results in higher overall precision for genetic variant analysis [73].

Finally, we obtained comprehensive and robust brain eQTLs by integrating multi-layer data from different resources. In particular, Hi-C data can explain how GWAS SNPs within enhancer regions could be related to their target genes, even when these genes are far from the SNPs. Considering the fact that most GWAS signals are in non-coding regions and SNPs within enhancer regions could act as eQTLs affecting their distal target genes, it is crucial to annotate and analyze eQTLs in enhancer regions by linking to their target genes. Our integrative approach of multi-layer of data enabled us to gain a comprehensive and robust resource for brain eQTLs in regulatory regions that could affect gene expression.

Some general limitations should be noted in here. First, while we employed several independent datasets, our cohorts do not have RNA-seq data, requiring reliance on other datasets with a larger sample size to study suicide risk in the context of gene expression regulation. In addition, the Korean mental health RNA-seq data was obtained from whole-blood samples from the Korean population which is not matched for ancestry with our sample. However, since other psychiatric disorders show associations with suicide risk molecular mechanisms, there is considerable appeal in studying the datasets to potentially find evidence of molecular mechanisms of genes identified by our gene-based analysis involved in suicide risk via the expression regulation of these related conditions. Second, in our main genomic analysis of WGS suicide deaths and general population controls, a relatively small number of local ancestry-matched control samples were analyzed compared to suicide deaths. Third, while analyses were restricted to suicide deaths and controls of European ancestry and analyses included residual ancestry principal components, residual effects of population stratification are possible. In particular, detailed demographic and clinical information was not available for either of the control datasets, limiting our ability to adjust for possible important covariates and preventing detailed analyses of diagnostic information. For gnomAD in particular, only aggregated allele frequency data are available. Finally, our results cannot address genetic risks in populations with non-European ancestry diversity.

In conclusion, pending replication, our integrative analyses present novel risk variants and genes associated with suicide death and their putative roles, showing convergent lines of evidence of risk for suicide death from comprehensive multi-layer of data in diverse independent resources. Our robust results may provide useful resources for future genetic discoveries, with the hope for eventual development of therapeutic targets and effective personalized preventative strategies.

### Supplementary information

Supplementary Figures

Supplementary Tables

## Data Availability

DNA aliquots from the Utah suicide deaths and associated phenotypic data are shared with the NIMH Biorepository (RUCDR) and the NIH Data Archive (NDA). Some other datasets investigated in this study are available from the following sources. The gnomAD v3.1.2 database: https://gnomad.broadinstitute.org/. GTEx database v8: https://gtexportal.org/home. ENCODE database: https://www.encodeproject.org/. Hi-C dataset in 3D Genome Browser: http://3dgenome.fsm.northwestern.edu/. Transcriptomic dataset of PsychENCODE: http://resource.psychencode.org/. Transcriptomic dataset of suicide attempter and major depressive disorder: SRP200298 in the NCBI database. Transcriptomic datasets of suicide death: GSE66937 and GSE101521 in the NCBI database. Additional data from this study are available from the authors upon request.

## Code availability

PLINK v1.9: https://www.cog-genomics.org/plink/1.9; BWA-MEM: https://maq.sourceforge.net/; GATK: https://gatk.broadinstitute.org/hc/en-us; kgp2anc: https://github.com/freeseek/kgp2anc; Sherlock: http://sherlock.ucsf.edu/.
