# Supplementary material for: Whole-genome sequencing analysis of suicide deaths integrating brain-regulatory eQTLs data to identify risk loci and genes
Source: Mol Psychiatry. 2023 Oct 4;28(9):3909–19. doi: 10.1038/s41380-023-02282-x (PMC10730410; doi:10.1038/s41380-023-02282-x)
Supplement: Supplementary file 1 — Supplementary Figures [file 41380_2023_2282_MOESM1_ESM.pdf]

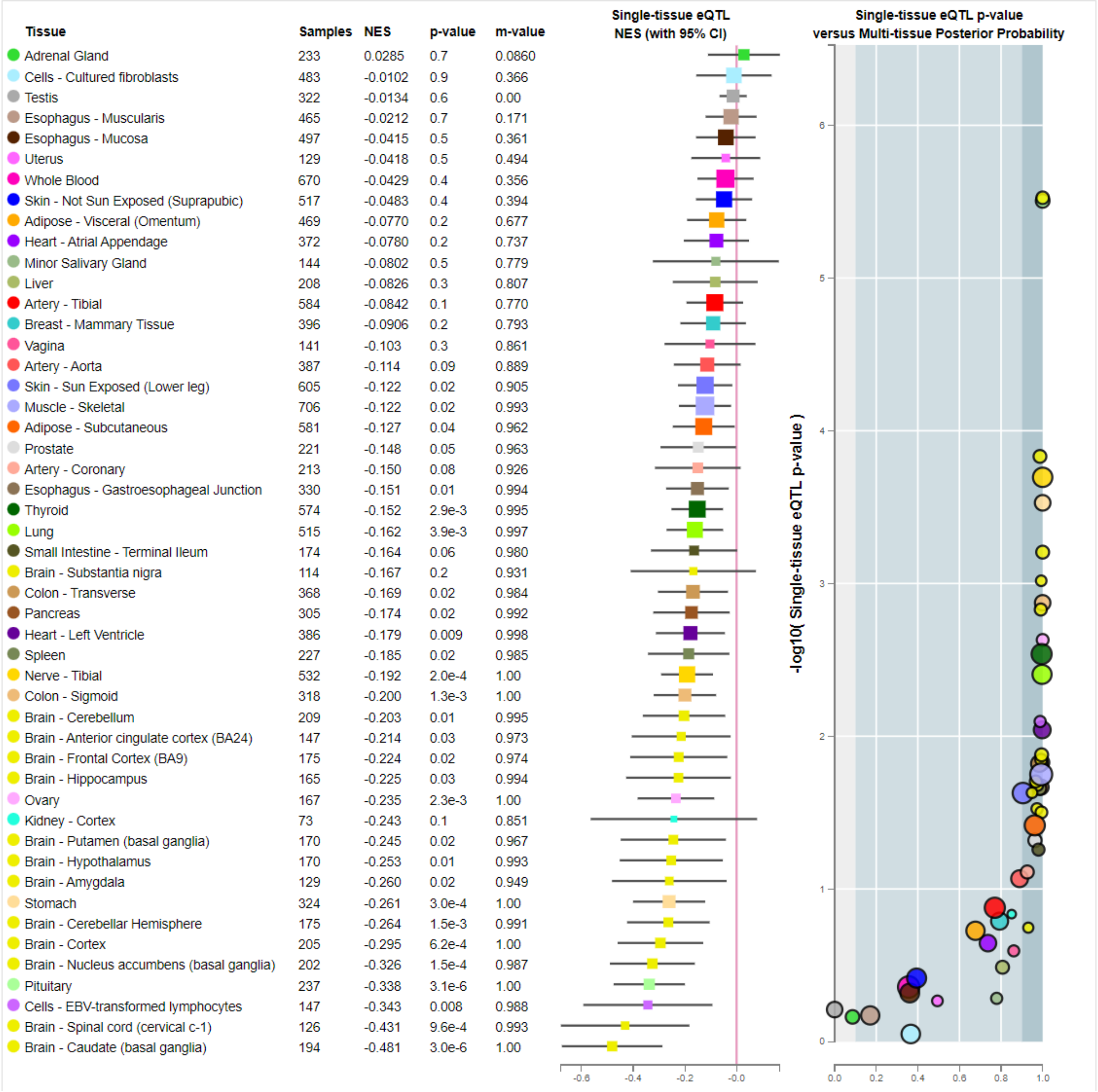

ENSG00000205853.10 RFPL3S and chr22_32385435_G_T_b38 eQTL (Meta Analysis RE2 P-Value: 6.345249999999999e-45)

Tissue	Samples	NES	p-value	m-value
Adrenal Gland	233	0.0285	0.7	0.0860
Cells - Cultured fibroblasts	483	-0.0102	0.9	0.366
Testis	322	-0.0134	0.6	0.00
Esophagus - Muscularis	465	-0.0212	0.7	0.171
Esophagus - Mucosa	497	-0.0415	0.5	0.361
Uterus	129	-0.0418	0.5	0.494
Whole Blood	670	-0.0429	0.4	0.356
Skin - Not Sun Exposed (Suprapubic)	517	-0.0483	0.4	0.394
Adipose - Visceral (Omentum)	469	-0.0770	0.2	0.677
Heart - Atrial Appendage	372	-0.0780	0.2	0.737
Minor Salivary Gland	144	-0.0802	0.5	0.779
Liver	208	-0.0826	0.3	0.807
Artery - Tibial	584	-0.0842	0.1	0.770
Breast - Mammary Tissue	396	-0.0906	0.2	0.793
Vagina	141	-0.103	0.3	0.861
Artery - Aorta	387	-0.114	0.09	0.889
Skin - Sun Exposed (Lower leg)	605	-0.122	0.02	0.905
Muscle - Skeletal	706	-0.122	0.02	0.993
Adipose - Subcutaneous	581	-0.127	0.04	0.962
Prostate	221	-0.148	0.05	0.963
Artery - Coronary	213	-0.150	0.08	0.926
Esophagus - Gastroesophageal Junction	330	-0.151	0.01	0.994
Thyroid	574	-0.152	2.9e-3	0.995
Lung	515	-0.162	3.9e-3	0.997
Small Intestine - Terminal Ileum	174	-0.164	0.06	0.980
Brain - Substantia nigra	114	-0.167	0.2	0.931
Colon - Transverse	368	-0.169	0.02	0.984
Pancreas	305	-0.174	0.02	0.992
Heart - Left Ventricle	386	-0.179	0.009	0.998
Spleen	227	-0.185	0.02	0.985
Nerve - Tibial	532	-0.192	2.0e-4	1.00
Colon - Sigmoid	318	-0.200	1.3e-3	1.00
Brain - Cerebellum	209	-0.203	0.01	0.995
Brain - Anterior cingulate cortex (BA24)	147	-0.214	0.03	0.973
Brain - Frontal Cortex (BA9)	175	-0.224	0.02	0.974
Brain - Hippocampus	165	-0.225	0.03	0.994
Ovary	167	-0.235	2.3e-3	1.00
Kidney - Cortex	73	-0.243	0.1	0.851
Brain - Putamen (basal ganglia)	170	-0.245	0.02	0.967
Brain - Hypothalamus	170	-0.253	0.01	0.993
Brain - Amygdala	129	-0.260	0.02	0.949
Stomach	324	-0.261	3.0e-4	1.00
Brain - Cerebellar Hemisphere	175	-0.264	1.5e-3	0.991
Brain - Cortex	205	-0.295	6.2e-4	1.00
Brain - Nucleus accumbens (basal ganglia)	202	-0.326	1.5e-4	0.987
Pituitary	237	-0.338	3.1e-6	1.00
Cells - EBV-transformed lymphocytes	147	-0.343	0.008	0.988
Brain - Spinal cord (cervical c-1)	126	-0.431	9.6e-4	0.993
Brain - Caudate (basal ganglia)	194	-0.481	3.0e-6	1.00

Supplementary Figure S1. The eQTL significance of rs926308 SNP for *RFPL3S* across to the multiple tissues. The SNP is significantly associated with *RFPL3S* expression (yellow) in most of brain tissues, but not in other tissues.

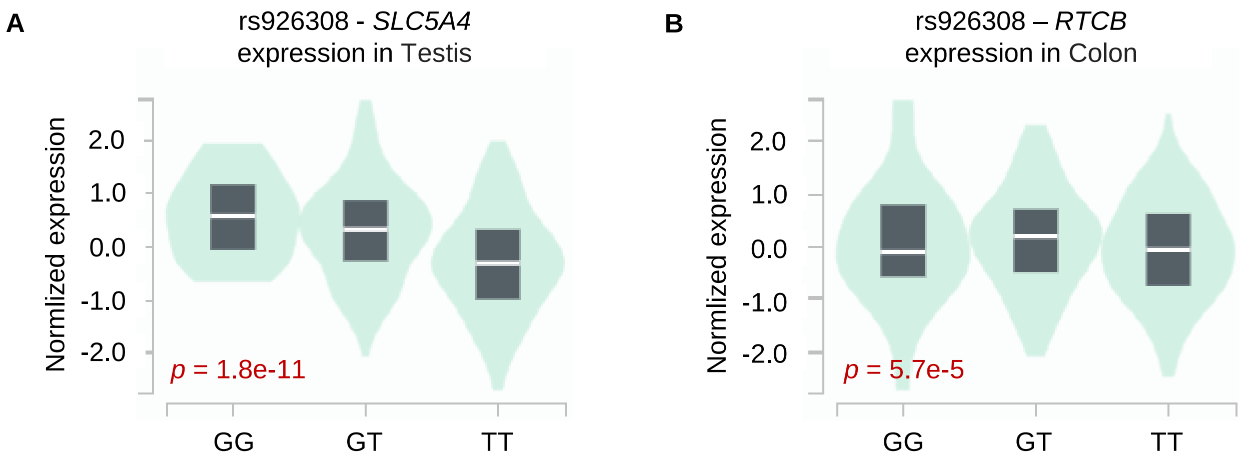

Supplementary Figure S2. The eQTL significance of rs926308, chr22:32385435, for (A) *SLC5A4* and (B) *RTCB* in testis and colon, respectively.
