# Supplementary material for: Whole-genome sequencing analysis of suicide deaths integrating brain-regulatory eQTLs data to identify risk loci and genes
Source: Mol Psychiatry. 2023 Oct 4;28(9):3909–19. doi: 10.1038/s41380-023-02282-x (PMC10730410; doi:10.1038/s41380-023-02282-x)
Supplement: Supplementary file 2 — Supplementary Tables [file 41380_2023_2282_MOESM2_ESM.pdf]

**Supplementary Table S1.** The comprehensive brain eQTLs of 13 brain tissues in regulatory regions from an integrative resource of GTEx, ENCODE, and 3D genome browser databases

| | GTEx | | | ENCODE | HI-C 3D genome browser |
|---|---|---|---|---|---|
| | # of samples (Male/Female) | # of eQTLs | # of eGenes | # of eQTLs in peaks from histone modification CHiP-seq, DNAase-seq & ATAC-seq | # of eQTLs in loop interactive & ENCODE peak regions |
| Amygdala | 129 (92/37) | 214115 | 3179 | 130403 | 150996 |
| Anterior cingulate cortex | 147 (105/42) | 314810 | 4710 | 194179 | 225860 |
| Caudate (basal ganglia) | 194 (142/52) | 505983 | 7298 | 310616 | 369432 |
| Cerebellar Hemisphere | 175 (124/51) | 585496 | 8828 | 361564 | 435846 |
| Cerebellum | 209 (151/58) | 709249 | 10117 | 441128 | 532587 |
| Cortex | 205 (141/64) | 573136 | 7980 | 351440 | 418066 |
| Frontal Cortex | 175 (127/48) | 441259 | 6324 | 270184 | 319408 |
| Hippocampus | 165 (116/49) | 324270 | 4696 | 200326 | 235537 |
| Hypothalamus | 170 (123/47) | 333680 | 4647 | 203878 | 240018 |
| Nucleus accumbens (basal ganglia) | 202 (147/55) | 498384 | 7020 | 302444 | 361490 |
| Putamen (basal ganglia) | 170 (128/42) | 413448 | 6033 | 256425 | 300754 |
| Spinal cord cervical | 126 (78/48) | 258477 | 3916 | 158242 | 187223 |
| Substantia nigra | 114 (81/33) | 175133 | 2812 | 107454 | 126604 |
| All brain regions[a] | | 1206469 | 17976 | 717852 | 898464 |
| Overlaped eSNPs in ENCODE and HI-C data | | | | **571773**[b] | |

[a] Union number of all brain regions, not number from mega- or meta-analysis

[b] The final comprehensive brain eQTLs in regulatory regions (histone modification and chromatin loop regions) for genomic association analysis

**Supplementary Table S2.** Summary of cohorts for differential expression analysis

| Cohort | ASD study[a] in Psychencode | | BD and SCZ study[b] in Psychencode | | | KMH[c] | | | SD dataset 1[d] | | SD dataset 2[e] | |
|---|---|---|---|---|---|---|---|---|---|---|---|---|
| Affection status | ASD | Control | BD | SCZ | Control | MDD[f] | SA[g] | Control | SD | Control | SD | Control |
| N | 43 | 65 | 145 | 346 | 559 | 39 | 56 | 87 | 39 | 28 | 21 | 29 |
| Female, n (%) | < 11 (< 25.58%) | 17 (26.15%) | 61 (42.06%) | 112 (32.36%) | 202 (36.14%) | 18 (46.15%) | 30 (53.57%) | 44 (50.57%) | - | - | < 11 (< 52.38%) | < 11 (< 37.93%) |
| Age, mean (SD) | 25.69 (17.33) | 27.76 (17.42) | 45.33 (13.73) | 60.45 (19.21) | 63.55 (23.74) | 32.10 (11.54) | 31.44 (10.99) | 25.28 (3.54) | - | - | 52.04 (21.74) | 43.52 (21.26) |

[a] Autism spectrum disorder (ASD) from UCLA-ASD and Yale-ASD projects in Psychencode

[b] Bipolar disorder (BD) and Schizophrenia (SCZ) from BrainGVEX, CommonMind Consortium (CMC), and CMC-HBCC projects in Psychencode

[c] Korean Mental Health

[d] Dataset 1 for suicide death (GEO: GSE66937). This data includes 10 suicide deaths and 7 controls for each of three brain regions: amygdala, prefrontal cortex, thalamus, and 9 suicide deaths and 7 controls for hippocampus region.

[e] Deatset 2 for 21 suicide deaths and 29 controls (GEO: GSE101521)

[f] Major depressive disorder

[g] Suicide attempter

**Supplementary Table S3.** The number of eQTLs in multiple brain regions investigated in whole-genome sequencing analysis

| Brain regions | # of eQTLs | % of eQTLs |
|---|---|---|
| Amygdala | 74699 | 16.96 |
| Anterior cingulate cortex | 113785 | 25.84 |
| Caudate (basal ganglia) | 187168 | 42.51 |
| Cerebellar Hemisphere | 221906 | 50.4 |
| Cerebellum | 271401 | 61.64 |
| Cortex | 210862 | 47.89 |
| Frontal Cortex | 161424 | 36.66 |
| Hippocampus | 118414 | 26.89 |
| Hypothalamus | 120099 | 27.28 |
| Nucleus accumbens (basal ganglia) | 180848 | 41.07 |
| Putamen (basal ganglia) | 153592 | 34.88 |
| Spinal cord cervical | 93192 | 21.16 |
| Substantia nigra | 62054 | 14.09 |
| Multiple brain regions[a] | 309637 | 70.32 |
| All brain regions[b] | 440324 | 100 |

[a] Union number of two or more than brain regions

[b] Union number of all brain regions

**Supplementary Table S4.** *Sherlock* integrative analysis result

| Genes | Top signal eSNP[a] | Chr | Position | Ref[b] | Alt[c] | LBF[d] | $P_{Sherlock}$ [e] | $P_{eQTL}$ [f] (direction[g]) | $P_{WGS}$ [h] (direction[i]) | $P_{Array}$ [j] (direction) |
|---|---|---|---|---|---|---|---|---|---|---|
| CNN3 | rs9432595 | 1 | 94860164 | G | A | 2.96927 | 4.51E-03 | 3.00E-13 (+) | 1.99E-03 (+) | 6.67E-04 (+) |
| NBL1 | rs12073300 | 1 | 19616437 | A | G | 3.05654 | 3.99E-03 | 1.90E-09 (-) | 2.53E-03 (-) | 7.85E-04 (-) |
| KIAA1143 | rs11922470 | 3 | 44736303 | C | T | 4.68668 | 3.51E-04 | 6.98E-15 (-) | 8.34E-05 (-) | 2.57E-04 (-) |
| MPRIPP1 | rs11922470 | 3 | 44736303 | C | T | 4.61402 | 4.47E-04 | 2.60E-06 (+) | 8.34E-05 (-) | 2.57E-04 (-) |
| PDCD6IP | rs9816877 | 3 | 33859810 | C | T | 3.66743 | 1.60E-03 | 1.80E-25 (+) | 6.67E-04 (+) | 3.23E-03 (+) |
| ZNF501 | rs4234448 | 3 | 44725387 | G | C | 3.86246 | 1.31E-03 | 3.30E-10 (+) | 2.33E-04 (-) | 4.56E-04 (-) |
| ZNF502 | rs7640654 | 3 | 44721545 | A | C | 3.40143 | 2.37E-03 | 4.91E-06 (+) | 1.07E-03 (-) | 5.79E-03 (-) |
| TBCA | rs2662352 | 5 | 77707470 | A | G | 3.6375 | 1.66E-03 | 3.23E-06 (-) | 4.12E-04 (-) | 3.21E-04 (-) |
| LEMD2 | rs6929696 | 6 | 33791513 | T | C | 3.04503 | 4.07E-03 | 2.59E-08 (-) | 3.71E-03 (+) | 1.75E-03 (+) |
| BCAP29 | rs7785892 | 7 | 107293730 | T | C | 3.14162 | 3.57E-03 | 1.7E-05 (-) | 1.80E-03 (-) | 3.63E-03 (-) |
| SLC18A2 | rs363224 | 10 | 117263062 | C | A | 3.00526 | 4.45E-03 | 1.99E-06 (-) | 4.35E-03 (+) | 2.26E-04 (+) |
| GDI2 | rs2797496 | 10 | 5759269 | T | G | 4.81967 | 3.93E-04 | 2.87E-06 (-) | 3.06E-04 (+) | 2.05E-03 (+) |
| SNX19 | rs7925664 | 11 | 130894631 | C | T | 4.4107 | 4.02E-04 | 4.30E-08 (+) | 5.80E-05 (+) | 3.56E-04 (+) |
| CBLN3 | rs17795094 | 14 | 24432227 | C | T | 3.08073 | 4.23E-03 | 6.74E-07 (-) | 3.14E-04 (+) | 2.26E-03 (+) |
| IGF1R | rs9672254 | 15 | 98954856 | C | T | 3.79068 | 1.43E-03 | 6.7E-19 (+) | 9.25E-05 (+) | 3.42E-04 (+) |
| KLHL36 | rs12444911 | 16 | 84658217 | C | T | 2.99586 | 4.48E-03 | 4.4E-09 (-) | 3.78E-03 (-) | 1.53E-03 (-) |
| KRT23 | rs7209663 | 17 | 40939055 | T | C | 3.93069 | 1.19E-03 | 1.40E-06 (+) | 1.53E-03 (-) | 2.19E-04 (-) |
| PCP4 | rs741792 | 21 | 39882224 | A | G | 4.72711 | 3.58E-04 | 6.69E-07 (-) | 1.76E-04 (-) | 6.07E-05 (-) |
| ARSA | rs6151429 | 22 | 50625049 | T | C | 2.8686 | 4.53E-03 | 4.40E-14 (-) | 3.81E-03 (-) | 9.53E-04 (-) |
| RFPL3S | rs5998471 | 22 | 32390230 | G | A | 4.06372 | 9.71E-04 | 5.59E-06 (-) | 9.61E-04 (+) | 3.89E-06 (+) |

[a] Top eSNP affecting target gene expression

[b] Reference allele

[c] Alternative allele

[d] LBF; logarithm Bayes factor, the larger LBF score means higher probability of gene expression-suicide relationship; for example, from the Sherlock analysis, the *SNX19* gene affected by rs7925664 was found. The Sherlock LBF score was 4.41 of SNX19 which means that this gene expression affected by rs7925664 change was estimated to be 82 times (exponential of 4.175) more likely to be associated with suicide than not.

[e] *P*-value from *Sherlock*

[f] *P*-value of eQTLs from GTEx

[g] Direction for expression or suicide risk of alternative allele

[h] *P*-value of our WGS data analysis

[i] Direction for suicide risk of alternative allele; +: risk allele and -: protective allele

[j] *P*-value of our Array data analysis

**Supplementary Table S5.** Expression analysis of psychiatric diseases for genes identified by *Sherlock* integrative analysis

| Gene | *P* | *Beta* | Psychiatric diseases | Dataset |
|---|---|---|---|---|
| ZNF501 | 7.64E-03 | -0.82544881 | Autism Spectrum Disorder | Psychencode |
| ARSA | 3.62E-04 | 0.920325898 | Bipolar Disorder | Psychencode |
| BCAP29 | 2.34E-04 | 0.448747344 | Schizophrenia | Psychencode |
| CNN3 | 1.59E-06 | 0.446113693 | Schizophrenia | Psychencode |
| IGF1R | 1.81E-07 | 0.535714478 | Schizophrenia | Psychencode |
| PDCD6IP | 5.55E-10 | 0.663913071 | Schizophrenia | Psychencode |
| SNX19 | 1.65E-04 | 0.363474854 | Schizophrenia | Psychencode |
| ARSA | 4.50E-03 | -0.402027495 | Schizophrenia | Psychencode |
| NBL1 | 2.46E-12 | -0.792621755 | Schizophrenia | Psychencode |
| IGF1R | 1.59E-04 | 0.124142681 | Suicide Attempter | KMH[a] |
| KLHL36 | 2.91E-03 | 0.051661714 | Suicide Attempter | KMH |
| PDCD6IP | 5.43E-03 | 0.013191823 | Suicide Attempter | KMH |
| SNX19 | 1.48E-03 | 0.0421164 | Suicide Attempter | KMH |

[a] Korean Mental Health

**Supplementary Table S6.** Expression analysis of suicide deaths for genes identified by *Sherlock* integrative analysis

| Gene | *P* | *Beta* | Empirical *P* [a] | Dataset |
|---|---|---|---|---|
| BCAP29 | 0.001105162 | -5.586553581 | 0.001 | Suicide death dataset 1 |
| SNX19 | 0.02208086 | 4.245444105 | 0.02 | Suicide death dataset 1 |
| KLHL36 | 0.028794109 | 3.813582446 | 0.032 | Suicide death dataset 1 |
| IGF1R | 0.000743515 | 8.017666738 | 0 | Suicide death dataset 1 |
| ZNF501 | 0.048800053 | -5.198998984 | 0.047 | Suicide death dataset 1 |
| ZNF502 | 0.00437428 | -5.735740329 | 0.005 | Suicide death dataset 1 |
| ZNF501 | 0.032192119 | -1.013970765 | 0.025 | Suicide death dataset 2 |

[a] Empirical *P*-value from 1,000 random permutation

**Supplementary Table S7.** Expression analysis of genes, stratified by sex

| Gene | All | | Male | | Female | | Psychiatric diseases | Significant group |
|---|---|---|---|---|---|---|---|---|
| | ***P*** | **Beta** | ***P*** | **Beta** | ***P*** | **Beta** | | |
| ARSA | 0.0003617 | 0.9203259 | 0.0009284 | 1.1117492 | 0.1373493 | 0.6142453 | Bipolar Disorder | Male |
| ARSA | 0.0535139 | 0.0247581 | 0.0032555 | 0.0572049 | 0.4141864 | -0.017944 | Major Depression Disorder | Male |
| IGF1R | 0.1118263 | 0.0746927 | 0.0016488 | 0.2277537 | 0.0848099 | -0.194638 | Major Depression Disorder | Male |
| SNX19 | 0.0001654 | 0.3634749 | 0.0006414 | 0.4376212 | 0.0800253 | 0.260184 | Schizophrenia | Male |
| LEMD2 | 0.0291408 | -0.288272 | 0.7365327 | -0.05601 | 0.0020657 | -0.697235 | Schizophrenia | Female |
| PCP4 | 0.4972456 | 0.2597168 | 0.1018203 | 0.0691152 | 0.0003044 | 0.1522166 | Suicide Attempter | Female |

**Supplementary Table S8.** Demographic information of suicide deaths with and without each of identified one SNP from SNP-based analysis and ten SNPs from gene-based analysis

| SNP | Chr | Position | Ref[a] | Alt[b] | | WGS samples | | Array samples | |
|---|---|---|---|---|---|---|---|---|---|
| | | | | | | Cases without alt allele of SNPs | Cases with alt allele of SNPs | Cases without alt allele of SNPs | Cases with alt allele of SNPs |
| rs9432595 | 1 | 94860164 | G | A | Mean age at death (SD) | 32.3 (13.28) | 32.15 (13.52) | 39.4 (17.13) | 40.84 (17.68) |
| | | | | | Female | 0.24 | 0.33 | 0.21 | 0.24 |
| | | | | | Male | 0.76 | 0.67 | 0.79 | 0.76 |
| rs12073300 | 1 | 19616437 | A | G | Mean age at death (SD) | 32.05 (13.3) | 32.06 (14.11) | 39.84 (17.2) | 40.58 (18.08) |
| | | | | | Female | 0.28 | 0.27 | 0.23 | 0.21 |
| | | | | | Male | 0.72 | 0.73 | 0.77 | 0.79 |
| rs7640654 | 3 | 44721545 | A | C | Mean age at death (SD) | 31.85 (13.7) | 32.12 (13.29) | 40.35 (17.69) | 39.84 (17.25) |
| | | | | | Female | 0.35 | 0.25 | 0.23 | 0.22 |
| | | | | | Male | 0.65 | 0.75 | 0.77 | 0.78 |
| rs4234448 | 3 | 44725387 | G | C | Mean age at death (SD) | 31.74 (13.17) | 32.29 (13.47) | 40.43 (17.59) | 39.85 (17.31) |
| | | | | | Female | 0.33 | 0.26 | 0.23 | 0.22 |
| | | | | | Male | 0.67 | 0.74 | 0.77 | 0.78 |
| rs9816877 | 3 | 33859810 | C | T | Mean age at death (SD) | 32.38 (13.44) | 32.64 (13.51) | 40.55 (17.41) | 39.4 (17.31) |
| | | | | | Female | 0.27 | 0.29 | 0.22 | 0.23 |
| | | | | | Male | 0.73 | 0.71 | 0.78 | 0.77 |
| rs7785892 | 7 | 107293730 | T | C | Mean age at death (SD) | 32.61 (13.28) | 31.24 (13.79) | 39.92 (17.23) | 40.13 (17.73) |
| | | | | | Female | 0.29 | 0.27 | 0.22 | 0.22 |
| | | | | | Male | 0.71 | 0.73 | 0.78 | 0.78 |
| rs7925664 | 11 | 130894631 | C | T | Mean age at death (SD) | 33.43 (13.96) | 31.94 (13.36) | 40.29 (17.35) | 39.93 (17.37) |
| | | | | | Female | 0.29 | 0.28 | 0.23 | 0.22 |
| | | | | | Male | 0.71 | 0.72 | 0.77 | 0.78 |
| rs9672254 | 15 | 98954856 | C | T | Mean age at death (SD) | 31.89 (13.18) | 32.16 (13.5) | 40.39 (17.87) | 39.83 (17.18) |
| | | | | | Female | 0.27 | 0.28 | 0.22 | 0.22 |
| | | | | | Male | 0.73 | 0.72 | 0.78 | 0.78 |
| rs12444911 | 16 | 84658217 | C | T | Mean age at death (SD) | 31.81 (13.09) | 32.13 (13.6) | 39.61 (17.22) | 40.3 (17.49) |
| | | | | | Female | 0.3 | 0.26 | 0.23 | 0.22 |
| | | | | | Male | 0.7 | 0.74 | 0.77 | 0.78 |

| | | | | | | | | | |
|---|---|---|---|---|---|---|---|---|---|
| rs6151429 | 22 | 50625049 | T | C | Mean age at death (SD) | 31.97 (13.52) | 32.37 (12.47) | 39.75 (17.32) | 41.38 (17.62) |
| | | | | | Female | 0.28 | 0.29 | 0.22 | 0.21 |
| | | | | | Male | 0.72 | 0.71 | 0.78 | 0.79 |
| rs926308 | 22 | 32385435 | G | T | Mean age at death (SD) | 32.97 (13.32) | 32.02 (13.4) | 39.11 (17.55) | 40.05 (17.35) |
| | | | | | Female | 0.33 | 0.28 | 0.24 | 0.22 |
| | | | | | Male | 0.67 | 0.72 | 0.76 | 0.78 |

[a] Reference allele

[b] Alternative allele

**Supplementary Table S9.** Prevalence of EHR diagnoses for suicide deaths with and without identified one SNP from SNP-based analysis and ten SNPs from gene-based analysis

| | | | | | | WGS samples | | | | Array samples | | | |
|---|---|---|---|---|---|---|---|---|---|---|---|---|---|
| EMR phenotype | SNP | Chr | Position | Ref[a] | Alt[b] | *P* | OR | Cases without alt allele of SNPs | Cases with alt allele of SNPs | *P* | OR | Cases without alt allele of SNPs | Cases with alt allele of SNPs |
| Accidentental trauma | rs9432595 | 1 | 94860164 | G | A | 0.441 | 0.889288 | 0.736328125 | 0.7116883 | 0.179 | 0.920977 | 0.640485541 | 0.6207967 |
| | rs12073300 | 1 | 19616437 | A | G | 0.802 | 1.05157 | 0.720159151 | 0.7290323 | 0.09 | 0.878154 | 0.638253638 | 0.6072235 |
| | rs7640654 | 3 | 44721545 | A | C | 0.373 | 1.155277 | 0.70260223 | 0.7326121 | 0.735 | 0.977204 | 0.636150235 | 0.6310764 |
| | rs4234448 | 3 | 44725387 | G | C | 0.332 | 1.179844 | 0.696428571 | 0.7330447 | 0.764 | 0.978233 | 0.63618677 | 0.6314085 |
| | rs9816877 | 3 | 33859810 | C | T | 0.412 | 1.13541 | 0.708971554 | 0.7356608 | 0.939 | 1.004646 | 0.631666667 | 0.6332476 |
| | rs7785892 | 7 | 107293730 | T | C | 0.735 | 0.943003 | 0.729651163 | 0.7090909 | 0.799 | 0.9829 | 0.633565621 | 0.6294574 |
| | rs7925664 | 11 | 130894631 | C | T | 0.725 | 1.089578 | 0.715789474 | 0.7257282 | 0.128 | 1.146368 | 0.604026846 | 0.6365394 |
| | rs9672254 | 15 | 98954856 | C | T | 0.45 | 0.873404 | 0.744186047 | 0.7191489 | 0.198 | 1.09117 | 0.617322835 | 0.6379908 |
| | rs12444911 | 16 | 84658217 | C | T | 0.082 | 0.771117 | 0.749445676 | 0.6991525 | 0.283 | 0.937059 | 0.640674875 | 0.6253444 |
| | rs6151429 | 22 | 50625049 | T | C | 0.258 | 0.787741 | 0.7325 | 0.688 | 0.509 | 0.945013 | 0.63445896 | 0.620384 |
| | rs926308 | 22 | 32385435 | G | T | 0.58 | 0.83781 | 0.75862069 | 0.7201405 | 0.53 | 0.930603 | 0.648199446 | 0.6311457 |
| Alcohol | rs9432595 | 1 | 94860164 | G | A | 0.652 | 1.07071 | 0.287109375 | 0.2987013 | 0.792 | 0.981954 | 0.244912531 | 0.2421107 |
| | rs12073300 | 1 | 19616437 | A | G | 0.237 | 0.783232 | 0.297082228 | 0.2516129 | 0.641 | 0.960048 | 0.24506237 | 0.238149 |
| | rs7640654 | 3 | 44721545 | A | C | 0.304 | 1.186469 | 0.267657993 | 0.301391 | 0.658 | 1.034442 | 0.23943662 | 0.2453704 |
| | rs4234448 | 3 | 44725387 | G | C | 0.313 | 1.196005 | 0.258928571 | 0.2987013 | 0.944 | 1.005827 | 0.243190661 | 0.2439288 |
| | rs9816877 | 3 | 33859810 | C | T | 0.821 | 1.035551 | 0.284463895 | 0.2942643 | 0.017 | 0.849976 | 0.25875 | 0.2283633 |
| | rs7785892 | 7 | 107293730 | T | C | 0.058 | 0.705875 | 0.308139535 | 0.2318182 | 0.34 | 0.929378 | 0.24738676 | 0.2341085 |
| | rs7925664 | 11 | 130894631 | C | T | 0.539 | 1.165136 | 0.273684211 | 0.2924757 | 0.14 | 1.168154 | 0.219798658 | 0.2472209 |
| | rs9672254 | 15 | 98954856 | C | T | 0.265 | 1.222004 | 0.260465116 | 0.3007092 | 0.008 | 1.232485 | 0.216535433 | 0.2537529 |
| | rs12444911 | 16 | 84658217 | C | T | 0.102 | 0.783192 | 0.310421286 | 0.2648305 | 0.51 | 0.956292 | 0.248062016 | 0.240063 |
| | rs6151429 | 22 | 50625049 | T | C | 0.347 | 0.810243 | 0.29375 | 0.256 | 0.598 | 1.051867 | 0.242297264 | 0.2525849 |
| | rs926308 | 22 | 32385435 | G | T | 0.246 | 0.713068 | 0.362068966 | 0.2833724 | 0.595 | 0.935288 | 0.254847645 | 0.2428539 |
| | rs9432595 | 1 | 94860164 | G | A | 0.337 | 1.146415 | 0.5234375 | 0.5506494 | 0.137 | 1.094057 | 0.38557658 | 0.4081738 |
| | rs12073300 | 1 | 19616437 | A | G | 0.192 | 0.784351 | 0.533156499 | 0.4774194 | 0.156 | 0.896595 | 0.399428274 | 0.3747178 |
| | rs7640654 | 3 | 44721545 | A | C | 0.4 | 1.137674 | 0.505576208 | 0.5378671 | 0.318 | 0.935456 | 0.406885759 | 0.3903356 |

| | | | | | | | | | | | | | |
|---|---|---|---|---|---|---|---|---|---|---|---|---|---|
| Anxiety (non-trauma) | rs4234448 | 3 | 44725387 | G | C | 0.231 | 1.213767 | 0.486607143 | 0.5396825 | 0.327 | 0.931937 | 0.408560311 | 0.3909876 |
| | rs9816877 | 3 | 33859810 | C | T | 0.287 | 1.165061 | 0.509846827 | 0.5486284 | 0.624 | 0.971257 | 0.39875 | 0.3907455 |
| | rs7785892 | 7 | 107293730 | T | C | 0.278 | 0.838372 | 0.546511628 | 0.4863636 | 0.015 | 0.848431 | 0.405342625 | 0.3666667 |
| | rs7925664 | 11 | 130894631 | C | T | 0.087 | 1.483209 | 0.463157895 | 0.5339806 | 0.159 | 1.136119 | 0.369127517 | 0.3985017 |
| | rs9672254 | 15 | 98954856 | C | T | 0.155 | 0.791577 | 0.562790698 | 0.5120567 | 0.739 | 1.022677 | 0.391338583 | 0.3960739 |
| | rs12444911 | 16 | 84658217 | C | T | 0.855 | 0.9751 | 0.527716186 | 0.5233051 | 0.289 | 0.938671 | 0.402644779 | 0.3880362 |
| | rs6151429 | 22 | 50625049 | T | C | 0.75 | 1.066201 | 0.5225 | 0.544 | 0.335 | 0.920863 | 0.397337934 | 0.379616 |
| | rs926308 | 22 | 32385435 | G | T | 0.631 | 0.87062 | 0.568965517 | 0.5245902 | 0.381 | 0.907066 | 0.415512465 | 0.393094 |
| Autism spectrum disorders | rs9432595 | 1 | 94860164 | G | A | 0.39 | 0.550529 | 0.013232514 | 0.0074074 | 0.636 | 1.176192 | 0.0059988 | 0.0067204 |
| | rs12073300 | 1 | 19616437 | A | G | 0.626 | 0.5947 | 0.010191083 | 0.0061728 | 0.29 | 1.509581 | 0.005761135 | 0.008547 |
| | rs7640654 | 3 | 44721545 | A | C | 0.985 | 0.986627 | 0.010676157 | 0.0104012 | 0.181 | 0.624369 | 0.008569545 | 0.0054334 |
| | rs4234448 | 3 | 44725387 | G | C | 0.681 | 0.751221 | 0.012987013 | 0.0096685 | 0.068 | 0.519643 | 0.009884679 | 0.0052849 |
| | rs9816877 | 3 | 33859810 | C | T | 0.682 | 0.766041 | 0.012552301 | 0.0095694 | 0.105 | 0.565616 | 0.007848733 | 0.004705 |
| | rs7785892 | 7 | 107293730 | T | C | 0.701 | 0.736539 | 0.011204482 | 0.0087336 | 0.088 | 0.437723 | 0.007416564 | 0.0032873 |
| | rs7925664 | 11 | 130894631 | C | T | 0.993 | 9471838 | NA | 0.0116414 | 0.838 | 1.115491 | 0.005641749 | 0.0063825 |
| | rs9672254 | 15 | 98954856 | C | T | 0.39 | 2.496984 | 0.004424779 | 0.0109141 | 0.901 | 1.049642 | 0.006036217 | 0.0063804 |
| | rs12444911 | 16 | 84658217 | C | T | 0.934 | 0.948813 | 0.010706638 | 0.0101215 | 0.597 | 1.198764 | 0.005762582 | 0.0067499 |
| | rs6151429 | 22 | 50625049 | T | C | 0.992 | 9.99E-08 | 0.012062726 | NA | 0.202 | 0.393783 | 0.006902322 | 0.0025478 |
| | rs926308 | 22 | 32385435 | G | T | 0.093 | 0.25891 | 0.032786885 | 0.0090192 | 0.159 | 0.503905 | 0.012019231 | 0.0058252 |
| Bipolar Disorders | rs9432595 | 1 | 94860164 | G | A | 0.73 | 0.951417 | 0.4375 | 0.4233766 | 0.948 | 1.005619 | 0.138521956 | 0.1386446 |
| | rs12073300 | 1 | 19616437 | A | G | 0.635 | 0.913362 | 0.425729443 | 0.4064516 | 0.365 | 0.904617 | 0.140852391 | 0.1286682 |
| | rs7640654 | 3 | 44721545 | A | C | 0.007 | 1.540783 | 0.356877323 | 0.4513138 | 0.693 | 0.963498 | 0.141627543 | 0.1374421 |
| | rs4234448 | 3 | 44725387 | G | C | 0.008 | 1.563788 | 0.34375 | 0.4487734 | 0.853 | 0.981348 | 0.140077821 | 0.1381543 |
| | rs9816877 | 3 | 33859810 | C | T | 0.464 | 1.112599 | 0.415754923 | 0.4438903 | 0.863 | 0.985552 | 0.139166667 | 0.1379606 |
| | rs7785892 | 7 | 107293730 | T | C | 0.033 | 0.69576 | 0.447674419 | 0.35 | 0.051 | 0.826119 | 0.144599303 | 0.1224806 |
| | rs7925664 | 11 | 130894631 | C | T | 0.664 | 1.106629 | 0.421052632 | 0.4247573 | 0.349 | 1.131091 | 0.125838926 | 0.140406 |
| | rs9672254 | 15 | 98954856 | C | T | 0.99 | 1.002038 | 0.418604651 | 0.4212766 | 0.945 | 1.006587 | 0.137795276 | 0.1388568 |
| | rs12444911 | 16 | 84658217 | C | T | 0.011 | 0.698675 | 0.461197339 | 0.3855932 | 0.019 | 0.820723 | 0.151390789 | 0.1275089 |
| | rs6151429 | 22 | 50625049 | T | C | 0.129 | 1.360558 | 0.41375 | 0.488 | 0.655 | 1.054545 | 0.137786542 | 0.1432792 |
| | rs926308 | 22 | 32385435 | G | T | 0.879 | 0.956824 | 0.448275862 | 0.4238876 | 0.507 | 1.114934 | 0.127423823 | 0.1394923 |
| | rs9432595 | 1 | 94860164 | G | A | 0.31 | 1.160526 | 0.62890625 | 0.6571429 | 0.759 | 0.981977 | 0.500178508 | 0.4956027 |
| | rs12073300 | 1 | 19616437 | A | G | 0.095 | 0.7304 | 0.644562334 | 0.5741935 | 0.023 | 0.843965 | 0.506237006 | 0.4638826 |
| | rs7640654 | 3 | 44721545 | A | C | 0.098 | 1.294868 | 0.594795539 | 0.6537867 | 0.939 | 0.994971 | 0.499217527 | 0.4979745 |

| | | | | | | | | | | | | | |
|---|---|---|---|---|---|---|---|---|---|---|---|---|---|
| Depression | rs4234448 | 3 | 44725387 | G | C | 0.155 | 1.262626 | 0.589285714 | 0.6479076 | 0.492 | 0.952662 | 0.507782101 | 0.4956827 |
| | rs9816877 | 3 | 33859810 | C | T | 0.367 | 1.142877 | 0.621444201 | 0.6533666 | 0.08 | 0.903168 | 0.510833333 | 0.4854327 |
| | rs7785892 | 7 | 107293730 | T | C | 0.305 | 0.843906 | 0.651162791 | 0.5954545 | 0.803 | 0.983872 | 0.49941928 | 0.4953488 |
| | rs7925664 | 11 | 130894631 | C | T | 0.219 | 1.330777 | 0.589473684 | 0.6359223 | 0.484 | 1.063228 | 0.484899329 | 0.5002417 |
| | rs9672254 | 15 | 98954856 | C | T | 0.671 | 0.930844 | 0.641860465 | 0.6283688 | 0.242 | 1.079844 | 0.484251969 | 0.5034642 |
| | rs12444911 | 16 | 84658217 | C | T | 0.803 | 0.965071 | 0.636363636 | 0.6292373 | 0.962 | 1.002762 | 0.497948016 | 0.4986226 |
| | rs6151429 | 22 | 50625049 | T | C | 0.211 | 0.774633 | 0.64 | 0.592 | 0.064 | 0.856976 | 0.503820557 | 0.465288 |
| | rs926308 | 22 | 32385435 | G | T | 0.679 | 1.129174 | 0.620689655 | 0.6346604 | 0.669 | 1.047918 | 0.487534626 | 0.4991996 |
| Drug | rs9432595 | 1 | 94860164 | G | A | 0.703 | 0.947178 | 0.404296875 | 0.3896104 | 0.425 | 1.054028 | 0.274901821 | 0.2840145 |
| | rs12073300 | 1 | 19616437 | A | G | 0.699 | 0.930018 | 0.396551724 | 0.3806452 | 0.323 | 0.920079 | 0.281964657 | 0.2641084 |
| | rs7640654 | 3 | 44721545 | A | C | 0.244 | 1.197457 | 0.36802974 | 0.4095827 | 0.23 | 1.092648 | 0.265258216 | 0.2835648 |
| | rs4234448 | 3 | 44725387 | G | C | 0.311 | 1.180796 | 0.361607143 | 0.4054834 | 0.163 | 1.118082 | 0.260700389 | 0.2835942 |
| | rs9816877 | 3 | 33859810 | C | T | 0.634 | 1.070868 | 0.38512035 | 0.40399 | 0.899 | 0.991775 | 0.27875 | 0.2784919 |
| | rs7785892 | 7 | 107293730 | T | C | 0.327 | 0.850413 | 0.409883721 | 0.3590909 | 0.066 | 0.87277 | 0.286004646 | 0.2589147 |
| | rs7925664 | 11 | 130894631 | C | T | 0.729 | 1.082804 | 0.389473684 | 0.3944175 | 0.351 | 1.097251 | 0.261744966 | 0.2810536 |
| | rs9672254 | 15 | 98954856 | C | T | 0.289 | 0.841233 | 0.423255814 | 0.3858156 | 0.413 | 1.06222 | 0.269291339 | 0.2820439 |
| | rs12444911 | 16 | 84658217 | C | T | 0.398 | 0.88908 | 0.407982262 | 0.3834746 | 0.733 | 0.978079 | 0.281349749 | 0.2762692 |
| | rs6151429 | 22 | 50625049 | T | C | 0.599 | 0.898522 | 0.39625 | 0.376 | 0.5 | 0.93864 | 0.280749322 | 0.2658789 |
| | rs926308 | 22 | 32385435 | G | T | 0.34 | 0.76457 | 0.465517241 | 0.3934426 | 0.544 | 0.929477 | 0.293628809 | 0.2773839 |
| Impulse control disorders | rs9432595 | 1 | 94860164 | G | A | 0.382 | 1.157584 | 0.19140625 | 0.2155844 | 0.424 | 1.081012 | 0.102463406 | 0.1070874 |
| | rs12073300 | 1 | 19616437 | A | G | 0.121 | 0.68963 | 0.209549072 | 0.1548387 | 0.107 | 0.809841 | 0.108108108 | 0.0880361 |
| | rs7640654 | 3 | 44721545 | A | C | 0.61 | 1.098375 | 0.189591078 | 0.2040185 | 0.774 | 0.969522 | 0.105633803 | 0.1038773 |
| | rs4234448 | 3 | 44725387 | G | C | 0.056 | 1.490722 | 0.151785714 | 0.2092352 | 0.995 | 1.000725 | 0.10311284 | 0.1046951 |
| | rs9816877 | 3 | 33859810 | C | T | 0.036 | 1.433634 | 0.172866521 | 0.2294264 | 0.908 | 0.988895 | 0.103333333 | 0.1053985 |
| | rs7785892 | 7 | 107293730 | T | C | 0.122 | 0.727399 | 0.207848837 | 0.1636364 | 0.286 | 0.889114 | 0.107142857 | 0.0968992 |
| | rs7925664 | 11 | 130894631 | C | T | 0.667 | 0.891365 | 0.210526316 | 0.1941748 | 0.767 | 0.958157 | 0.105704698 | 0.1041566 |
| | rs9672254 | 15 | 98954856 | C | T | 0.756 | 1.063271 | 0.190697674 | 0.2 | 0.861 | 1.019248 | 0.102362205 | 0.1050808 |
| | rs12444911 | 16 | 84658217 | C | T | 0.081 | 0.747904 | 0.219512195 | 0.1737288 | 0.217 | 0.888044 | 0.110807114 | 0.09878 |
| | rs6151429 | 22 | 50625049 | T | C | 0.166 | 0.693201 | 0.20625 | 0.152 | 0.414 | 0.888941 | 0.106482623 | 0.0915805 |
| | rs926308 | 22 | 32385435 | G | T | 0.882 | 1.052669 | 0.189655172 | 0.1990632 | 0.512 | 1.130009 | 0.096952909 | 0.1049623 |
| | rs9432595 | 1 | 94860164 | G | A | 0.733 | 1.079687 | 0.09765625 | 0.1038961 | 0.22 | 0.836639 | 0.048197072 | 0.0403518 |
| | rs12073300 | 1 | 19616437 | A | G | 0.816 | 1.070421 | 0.096816976 | 0.1032258 | 0.768 | 0.947354 | 0.04547817 | 0.0428894 |
| | rs7640654 | 3 | 44721545 | A | C | 0.047 | 1.730436 | 0.066914498 | 0.1097372 | 0.492 | 1.117827 | 0.041471049 | 0.0462963 |

| | | | | | | | | | | | | | |
|---|---|---|---|---|---|---|---|---|---|---|---|---|---|
| Schizophrenia/ schizoaffective | rs4234448 | 3 | 44725387 | G | C | 0.126 | 1.554613 | 0.071428571 | 0.1082251 | 0.3 | 1.204032 | 0.038910506 | 0.0466811 |
| | rs9816877 | 3 | 33859810 | C | T | 0.554 | 1.146474 | 0.09190372 | 0.1047382 | 0.824 | 1.03175 | 0.044166667 | 0.045844 |
| | rs7785892 | 7 | 107293730 | T | C | 0.172 | 0.674128 | 0.10755814 | 0.0727273 | 0.43 | 0.880028 | 0.046457607 | 0.0410853 |
| | rs7925664 | 11 | 130894631 | C | T | 0.831 | 1.082274 | 0.094736842 | 0.098301 | 0.435 | 1.192166 | 0.038590604 | 0.0459159 |
| | rs9672254 | 15 | 98954856 | C | T | 0.888 | 0.963274 | 0.097674419 | 0.0950355 | 0.636 | 1.079282 | 0.042519685 | 0.0459007 |
| | rs12444911 | 16 | 84658217 | C | T | 0.351 | 0.811562 | 0.106430155 | 0.0889831 | 0.198 | 0.834878 | 0.049247606 | 0.0413223 |
| | rs6151429 | 22 | 50625049 | T | C | 0.802 | 1.082962 | 0.09625 | 0.104 | 0.152 | 0.7248 | 0.046832635 | 0.0339734 |
| | rs926308 | 22 | 32385435 | G | T | 0.56 | 0.781865 | 0.120689655 | 0.0948478 | 0.486 | 0.84179 | 0.052631579 | 0.0443631 |
| Suicidal ideation | rs9432595 | 1 | 94860164 | G | A | 0.631 | 1.073239 | 0.30078125 | 0.3142857 | 0.847 | 0.985464 | 0.189575152 | 0.1857217 |
| | rs12073300 | 1 | 19616437 | A | G | 0.359 | 0.833188 | 0.307692308 | 0.2709677 | 0.883 | 1.01415 | 0.187889813 | 0.1884876 |
| | rs7640654 | 3 | 44721545 | A | C | 0.268 | 1.196896 | 0.275092937 | 0.3122102 | 0.378 | 0.929306 | 0.195618153 | 0.1851852 |
| | rs4234448 | 3 | 44725387 | G | C | 0.184 | 1.259372 | 0.263392857 | 0.3131313 | 0.496 | 0.940803 | 0.194552529 | 0.1861846 |
| | rs9816877 | 3 | 33859810 | C | T | 0.691 | 1.061331 | 0.297592998 | 0.3117207 | 0.925 | 1.007038 | 0.186666667 | 0.1893745 |
| | rs7785892 | 7 | 107293730 | T | C | 0.807 | 0.959035 | 0.306686047 | 0.2909091 | 0.909 | 0.990487 | 0.18844367 | 0.1868217 |
| | rs7925664 | 11 | 130894631 | C | T | 0.839 | 1.049839 | 0.294736842 | 0.2985437 | 0.065 | 1.245276 | 0.159395973 | 0.1921218 |
| | rs9672254 | 15 | 98954856 | C | T | 0.586 | 1.0996 | 0.279069767 | 0.2992908 | 0.077 | 1.16477 | 0.170866142 | 0.1942841 |
| | rs12444911 | 16 | 84658217 | C | T | 0.625 | 0.931481 | 0.305986696 | 0.2923729 | 0.182 | 0.905156 | 0.196534428 | 0.1806375 |
| | rs6151429 | 22 | 50625049 | T | C | 0.18 | 1.315743 | 0.29125 | 0.352 | 0.474 | 0.925042 | 0.190041903 | 0.1757755 |
| | rs926308 | 22 | 32385435 | G | T | 0.118 | 0.643399 | 0.396551724 | 0.293911 | 0.822 | 0.969284 | 0.193905817 | 0.1875143 |
| Suicide attempt | rs9432595 | 1 | 94860164 | G | A | 0.198 | 1.274836 | 0.128544423 | 0.1580247 | 0.22 | 1.144583 | 0.062387522 | 0.0694444 |
| | rs12073300 | 1 | 19616437 | A | G | 0.095 | 0.624389 | 0.149044586 | 0.0987654 | 0.017 | 0.690798 | 0.069133614 | 0.048433 |
| | rs7640654 | 3 | 44721545 | A | C | 0.043 | 1.56223 | 0.106761566 | 0.1575037 | 0.249 | 1.156526 | 0.058668425 | 0.067671 |
| | rs4234448 | 3 | 44725387 | G | C | 0.089 | 1.503601 | 0.103896104 | 0.1491713 | 0.201 | 1.193396 | 0.056836903 | 0.0675551 |
| | rs9816877 | 3 | 33859810 | C | T | 0.325 | 1.210233 | 0.127615063 | 0.1507177 | 0.974 | 1.00358 | 0.064573671 | 0.0658704 |
| | rs7785892 | 7 | 107293730 | T | C | 0.745 | 0.930592 | 0.145658263 | 0.1353712 | 0.993 | 0.998905 | 0.06526576 | 0.0650888 |
| | rs7925664 | 11 | 130894631 | C | T | 0.569 | 0.847063 | 0.163265306 | 0.1396973 | 0.515 | 1.116761 | 0.059238364 | 0.0660902 |
| | rs9672254 | 15 | 98954856 | C | T | 0.913 | 1.024543 | 0.137168142 | 0.1405184 | 0.795 | 1.032659 | 0.063715627 | 0.0657669 |
| | rs12444911 | 16 | 84658217 | C | T | 0.519 | 1.127832 | 0.132762313 | 0.1477733 | 0.142 | 1.174764 | 0.060315021 | 0.0695241 |
| | rs6151429 | 22 | 50625049 | T | C | 0.98 | 0.993456 | 0.141133896 | 0.1407407 | 0.025 | 0.667567 | 0.068395733 | 0.0458599 |
| | rs926308 | 22 | 32385435 | G | T | 0.87 | 0.940702 | 0.147540984 | 0.1386697 | 0.347 | 0.833885 | 0.076923077 | 0.0642718 |

[a] Reference allele

[b] Alternative allele
